# Carbonate‐ Versus Hydroxide‐Based Precursor for the Synthesis of Li/Mn Rich Cathodes: Exploring Differences in Discharge Capacities and Cycle Life

**DOI:** 10.1002/smsc.70370

**Published:** 2026-08-12

**Authors:** Pranti Sutar, Johannes Helmut Thienenkamp, Laurin Profanter, Tim Messink, Annalena Krude, Uta Rodehorst, Hyuck Hur, Gunther Brunklaus, Dominik Voigt, Martin Winter, Johannes Kasnatscheew

**Affiliations:** ^1^ MEET Battery Research Center Institute of Physical Chemistry University of Münster Münster Germany; ^2^ Helmholtz‐Institute Münster IMD‐4 Forschungszentrum Jülich GmbH Münster Germany; ^3^ LG Energy Solution Seoul Republic of Korea

**Keywords:** cyclelife, Li^+^ extraction ratio, Li/Mn rich, lithiation capacity, Mn‐redox, precursor, specific surface area

## Abstract

Lithium/manganese‐rich (LMR)‐based cathodes can enhance the specific energy of Li ion batteries and are conventionally synthesized via a carbonate‐based precursor (LMR‐CO_3_). The CO_2_ evolution‐related voids and enhanced surface area can be avoided by calcination of a hydroxide‐based precursor [LMR‐(OH)_2_]. This way, the LMR releases less oxygen during charge, as seen by a decreased Mn^3+/4+^ redox activity and decreased voltage fade. However, the lower specific discharge capacity is attributed to a decreased lithiation amount in the initial cycles for LMR‐(OH)_2_ and is related to a higher Li^+^ extraction degree, that is, a higher redox oxidation number at the discharged state. Interestingly, this decreases the accompanying amount of detrimental Mn^3+^ and is concluded to improve cycle life, due to, for example, minimized Jahn–Teller distortions and disproportionation reactions. The differences in lithiation (e^−^ + Li^+^) degrees of the two LMRs emerge at end‐of‐discharge and can stem from differences in either redox resistance and/or resistance of Li^+^ intercalation, for example, due to slight crystal stacking faults in the course of different synthesis conditions and/or simply from bigger particle sizes.

## Graphical abstract

The synthesis of Li/Mn‐rich cathodes via hydroxide‐based precursor can be beneficial in terms of, for example, lower surface area. The apparently disadvantageous lower lithiation amount (discharge capacity) is shown to be beneficial for cycle life, as the higher redox oxidation number in the discharged state suggests a lower amount of detrimental Mn^3+^.

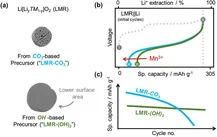

## 1 Introduction

Electrochemical energy storage technologies like lithium‐ion batteries (LIBs) can circumvent the dependence on fossil fuels by realizing storage of renewable energies in the form of electricity [1, 2]. However, the remaining challenges require further R&D, in particular on cathode active materials (CAM), which are a bottleneck in terms of costs and relevant performance aspects. Layered oxide‐based CAMs like Ni‐rich LiNi*_x_* Co*_y_* Mn*_z_* O_2_ (NCM; *x *+ *y *+ *z* = 1) are state‐of‐the‐art (SOTA) as they, among other advantages, provide a high practical specific capacity (up to ~200 mAh g^−1^)—depending on stoichiometry—and mean discharge voltage (~3.7 V) [3, 5]. For reasons of sustainability and costs, the focus on "low‐Co" and high‐energy CAMs progressively emerges. Here, Li‐ and Mn‐rich CAMs (Li[Li*_y_* TM_1−*y*_]O_2_; TM = Ni, Co, Mn; 0 < *y* < 1/3; abbreviated as LMR) are of particular interest because of their even higher practical specific capacity compared to NCM (>250 mAh g^−1^) at a still moderate discharge voltage (~3.5 V) [1, 6]. However, severe structural degradation, voltage decay, and excessive gassing during operation cause energy fade and safety issues [7, 8]. Among others, this can be attributed to irreversible oxygen release throughout cycling, which, for reasons of charge compensation, enhances Mn^3+/4+^ redox activity, which operates at lower voltages than the O^2−/0^ redox, leading to voltage fade and structural degradation [9, 10]. Besides the well‐known attempts to address these issues via surface coatings, doping and/or electrolyte additives [6, 11], the modification of the CAM particles and their morphology via altered synthesis approaches can also be viable.

Conventionally, LMRs are synthesized by carbonate‐based coprecipitation, that is, at a relatively low pH (pH 7–8) and low temperature for precursor synthesis, where the oxidation state of the TM cations is kept +2 for all TMs [12, 14]. Industrial practice commonly employs mixed TM‐carbonate precursors for LMR cathode manufacture, and suppliers provide Ni–Mn‐carbonate precursors for LMR‐CAM production [15, 16]. In contrast, the higher required pH for the precipitation with OH^‐^, including the risk of detrimental Mn‐oxide precipitation, renders this synthesis more challenging and sensitive. However, other than OH^−^‐, the CO_3_^2−^‐based LMR precursors tend to have higher CO_2_ evolution during high‐temperature sintering [17], which increases surface area and voids within the spherical secondary particle and consequently facilitates irreversible oxygen release, finally rendering replacement with hydroxides reasonable. Nevertheless, synthesis of LMRs with higher manganese content than nickel (here 65% Mn and 35% Ni) is challenging with hydroxide coprecipitation because it requires higher pH (pH > 10.5) and can enhance Mn‐based side products if reaction conditions are not optimized [14, 18].

In this work, discrepancies in discharge capacities between LMR synthesized from a carbonate‐based precursor (denoted as LMR‐CO_3_) and a hydroxide‐based precursor (denoted as LMR‐(OH)_2_) are thoroughly investigated and discussed.

## 2 Experimental Section

### 2.1 Material Synthesis

Carbonate‐based LMR CAM has been provided by LG Energy Solutions. For LMR‐(OH)_2_ CAM, the hydroxide precursor is synthesized via continuous Laminar Couette–Taylor flow reactor (LCTR) (Laminar, LCTR Terra 3300) using the coprecipitation method described by Lee et al., which is also commonly used in the case of synthesis of nickel‐rich layered oxides [19, 20]. Spherical precursor particles (Ni_0.35_Mn_0.65_)(OH)_2_ were synthesized by placing 1.5 (M) aqueous solution of 35% NiSO_4_·8H_2_O and 65% MnSO_4_·7H_2_O in a 5 L bottle, which was then pumped into the reactor at a constant flow rate. At the same time, 4.8 (M) aqueous solution of NaOH was also added to keep the reactant mixture at a constant pH during the entire reaction time. The desired amount of 12% NH_4_OH (W/W as NH_3_) solution (aq.) was also fed into the reactor in such a way that the ratio of TM:NH_4_OH = 1:1 at all times. The suitable pH for Mn‐rich hydroxide precursor was found to be 10.8, which is substantially lower than that used for the synthesis of the hydroxide precursor of Ni‐rich NCMs. This is because Mn tends to form undesired oxides at higher pH [18, 21]. The reaction mixture is heated at 70 °C throughout the entire reaction time. The LCTR reactor has a total volume of 1000 mL, and the mean residence time for a secondary particle to travel through the entire length of the reactor depends on the total flow rate of all the reactant solutions. At the beginning of the reaction, the particles are mostly agglomerated, and as the reaction proceeds, the Taylor vortices increase the force of mixing, promoting spherical precipitates with a narrow particle size distribution. After an equilibrium time of 18 h (four times the mean residence time of 4 h), the precipitate was collected in a bottle containing cold distilled (DI) water. The collected precipitates were washed multiple times with DI water to remove all the by‐products and also to bring the pH down to 9. The spherical (Ni_0.35_Mn_0.65_)(OH)_2_ precipitates were then filtered and dried in a vacuum oven at 80 °C overnight to remove all absorbed water. It is very important to dry the precursor with lower nickel content (>50%) in a vacuum oven instead of a normal oven because the growth of small particles has been observed on the surface of spherical secondary particles when the precursor was dried in a conventional oven.

The dried hydroxide precursor was mixed with LiOH·H_2_O in a molar ratio of LiOH: precursor = 1.35 thoroughly with a mortar and pestle. The sample mixture was rapidly heated (10 °C/min) up to 500 °C, held for 2 h, and then further heated at the same rate up to 950 °C for 12 h. After that, the mixture was slowly cooled down at a rate of 2 °C/min until it reached 50 °C or room temperature.

### 2.2 Material Characterization

Powder X‐ray diffraction (XRD, Bruker D8 Advance) was measured using Bragg–Brentano geometry from 5° to 90° at a step size of 0.02° s^−1^ with Cu K_α_ radiation (*λ *= 0.154 nm) at 40 kV and 40 mA using a divergence slit of 0.64 mm to identify the crystalline phases of the LMR powder Li_*x*_Ni_0.35_Mn_0.65_O_2_. With the LYNXEYE XE‐T detector, Fe fluorescence excited by Cu radiation was filtered 100% at zero intensity loss. For in situ calcination studies a nonambient temperature chamber from Bruker was used as the sample stage, and XRD was measured using Cu‐K_α_ radiation. Pawley and Rietveld refinements were performed to analyze the XRD data using the TOPAS program by Bruker.

For operando XRD studies, a modified design of the operando electrochemical cell was used in transmission mode using Mo‐K_α_ as the X‐ray source. The cell design is vastly adapted from Argonne’s multipurpose in situ X‐ray (AMPIX) [22] cells by Borkiewicz et al. from Argonne National Laboratory. Our operando cell design was inspired by the AMPIX cell but slightly modified to be more compatible with our setup. The cell was operated in a two‐electrode setup, versus metallic lithium, i.e., a half‐cell setup. The detailed cell design is given in the Supporting Information (S17).

The morphology of the powder sample was analyzed using scanning electron microscopy (SEM) using a Carl Zeiss AURIGA field emission microscope with a Schottky field emitter as an electron source and a typical accelerating voltage of 3 kV.

Inductively coupled plasma–optical emission spectroscopy (ICP–OES) was done with all LMRs to accurately determine the elemental composition and stoichiometric ratio of the TMs. The material was dissolved in aqua regia using a microwave digestion system, a Multiwave 7000 (AntonPaar, Graz, Austria). ICP–OES was performed using an ARCOS (Spectro Analytical Instruments GmbH) equipped with a Scott spray chamber, a cross‐flow nebulizer, and an axially positioned plasma torch. The following emission lines were used for quantification during analysis: Li (670.780 nm), Mn (257.611, 259.373, 403.076 nm), Ni (221.648, 231.604, 232.003 nm), and S (180.731 nm).

For thermogravimetric analysis (TGA), a TGA Q5000IR (TA Instruments) was used at a heating rate of 10 K min^−1^ in a temperature range between 30 and 950 °C using an alumina pan carried by a platinum high‐temperature pan and an oxygen sample gas flow of 25 mL min^−1^

Specific surface area of the LMR‐CAM samples was determined by the Brunauer–Emmett–Teller (BET) method [23] using krypton. The physisorption data were obtained using a 3 Flex device (Micromeritics GmbH) at a liquid nitrogen temperature of −196 °C. The CAM powders were vacuum dried at 120 °C overnight prior to measurements. The entire procedure was followed according to IUPAC guidelines reported by Thommes et al. [24].

Surface species were identified by X‐ray photoelectron spectroscopy (XPS). XPS measurements were carried out with powder LMR samples pressed into 12 mm pellets as well as pristine electrodes. Samples were mounted on a sample holder and transported to a glovebox connected to an Axis Ultra DLD XPS (Kratos Analytical). From here, samples were moved into an ultra‐high vacuum (*p* ≤ 10^−8^ mbar) chamber inside the device. XPS was measured using a monochromatic Al K_α_ source (*h* *ν* = 1486.6 eV) at an emission current of 10 mA with an accelerating voltage of 12 kV. A charge neutralizer was used to suppress positive charging of the sample surface. The area of analysis was approximately 700 x 300 μm. The angle of emission was 0° with respect to the surface normal, and the hemispherical analyzer was set to a pass energy of 20 eV. Core spectra were recorded in the following regions: F 1s, Mn 2p, Ni 2p, O 1s, C 1s, P 2p, and Li 1s. After the samples had been measured, the resulting data was evaluated with CasaXPS (v2.3.23, Casa Software) [25]. A Shirley‐type background was used to account for inelastically scattered photoelectrons.

The concentration of surface species like Li_2_CO_3_ and LiOH was also determined by Wader titration using a 905 Titrando autotitrator from Metrohm. For this, 0.625 g powder samples were stirred in 25 mL ultrapure water (Milipore) for 2 min in a nitrogen‐filled glove bag and afterward filtered through a frit. The filtrate was collected in a round‐bottom flask, which was flushed with Ar before 10 mL was transferred into an airtight titration vessel. Then 0.05 mol L^−1^ of HCl was added at a dynamic dosing rate under constant stirring. Each measurement was repeated twice for reproducibility. The equivalence point was determined from the differential curve of *d*(pH)/*d* *V* versus *V* (volume of HCl added). The method was described by Schuer et al. [26].

^7^Li Magic angle spinning (MAS) NMR spectra were acquired at a Bruker Avance III 4.7T NMR spectrometer equipped with a commercially available Bruker 1.3 mm double‐resonance (1H/X) MAS probe. A rotor‐synchronized Oldfield echo pulse program, a MAS rate of 55 kHz, and a π/2 pulse of 0.9 µs (=277.8 kHz excitation bandwidth) were applied, averaging 32 768 transients at 2 s recycle delay, thus resulting in a total experimental time of 18 h and 23 min per NMR spectrum. All the spectra were processed with the Bruker TopSpin 3.5pl7 software and referenced to 1 M LiCl (aq.). Subsequent spectral deconvolution was performed within the DmFit program package [27].

### 2.3 Electrode Preparation and Electrochemical Investigations

For electrochemical investigation, LMR || Li metal half cells were prepared. The positive electrode was cast with 92.50 wt% LMR active material, 3 wt% carbon black (Super C_65_, Imerys Graphite & Carbon), 4.50 wt% PVdF as binder (Solef 5130, Solvay). NMP (anhydrous, purity: 99.5%, Sigma‐Aldrich) was used with a total solid content of 60 wt%. The mixture was homogeneously mixed in a Thinky Mixture for 20 min. The electrode paste was coated with a doctor blade (Zehntner GmbH) on previously washed (with acetone) aluminum foil of thickness 20 μm in an automatic film applicator (Sheen Instruments). The electrode sheets were dried in a vacuum oven at 80 °C for 3–4 h and then punched out into 12 mm and dried in a Büchi glass drying oven at 120 °C under reduced pressure (~1 x 10^−3^ bar) for 12 h. The electrodes were calendared with a hydraulic press to reach a porosity of 30% before cell assembly. The average mass loading of the positive electrode was ≈6.8 ± 0.8 mg_LMR_ cm^−2^. Charge/discharge investigations were performed on a Maccor series 4000 battery tester (Maccor, Inc.) at 40 °C for the formation cycle and at 20 °C for capacity check and long‐term cycling performance, using coin cell (CR2032) and the electrolyte was 1.0 (M) LiPF_6_ in ethylene carbonate/ethyl methyl carbonate (3:7 by weight), with 2% LiBF_4_ and 5% fluoroethylene carbonate.

The formation cycle consists of one constant current (CC) cycle with upper and lower cut‐off voltages of 4.65 and 2.0 V with a C‐rate of 0.1 C and was performed at 40 °C. A constant voltage (CV) step was performed once the upper cut‐off voltage was reached during charging until the current reached 0.05 C. For long‐term cycling, the cycling procedure contains one cycle after formation with CCCV (0.1 C) during charging and CC (0.1 C) during discharge, followed by 800 cycles with CCCV (0.33 C) during charge and CC (0.33 C) during discharge, both at 20 °C. The galvanostatic intermittent titration (GITT) measurement was performed for LMRs versus Li metal cells. The occurring resistances, especially charge transfer (*R*_CT_) and diffusion resistance (*R*_D_) of the charge/discharge cycle, were determined in dependence on their state of charge (SoC). The cells were charged and discharged using 30 min C/10 current pulses, followed by a 4 h rest step until the voltage dropped and approached equilibrium. 65 such current pulses were applied until it reached 4.7 V during charge, and the same number of current pulses was applied during discharge until 1.5 V was reached. A simplified representation of such current pulses has been shown and described in the Supporting Information (Figure S9).

## 3 Results and Discussion

### 3.1 Morphology Comparison

The stoichiometries were determined via Li_1.15_(Ni_0.35_Mn_0.65_)_0.85_O_2_ and Li_1.13_(Ni_0.35_Mn_0.65_)_0.87_O_2_ for LMR‐CO_3_ and LMR‐(OH)_2,_ respectively, obtained by means of ICP–OES (Table S1). Given the different synthesis conditions, the Li amount can stem from the surface and slightly impact the ICP–OES data, thereby affecting the calculated stoichiometry. However, as the electrochemical data during initial charge and crystal characteristics are initially similar (discussed next), similar crystal stoichiometry can be concluded.

**FIGURE 1:**
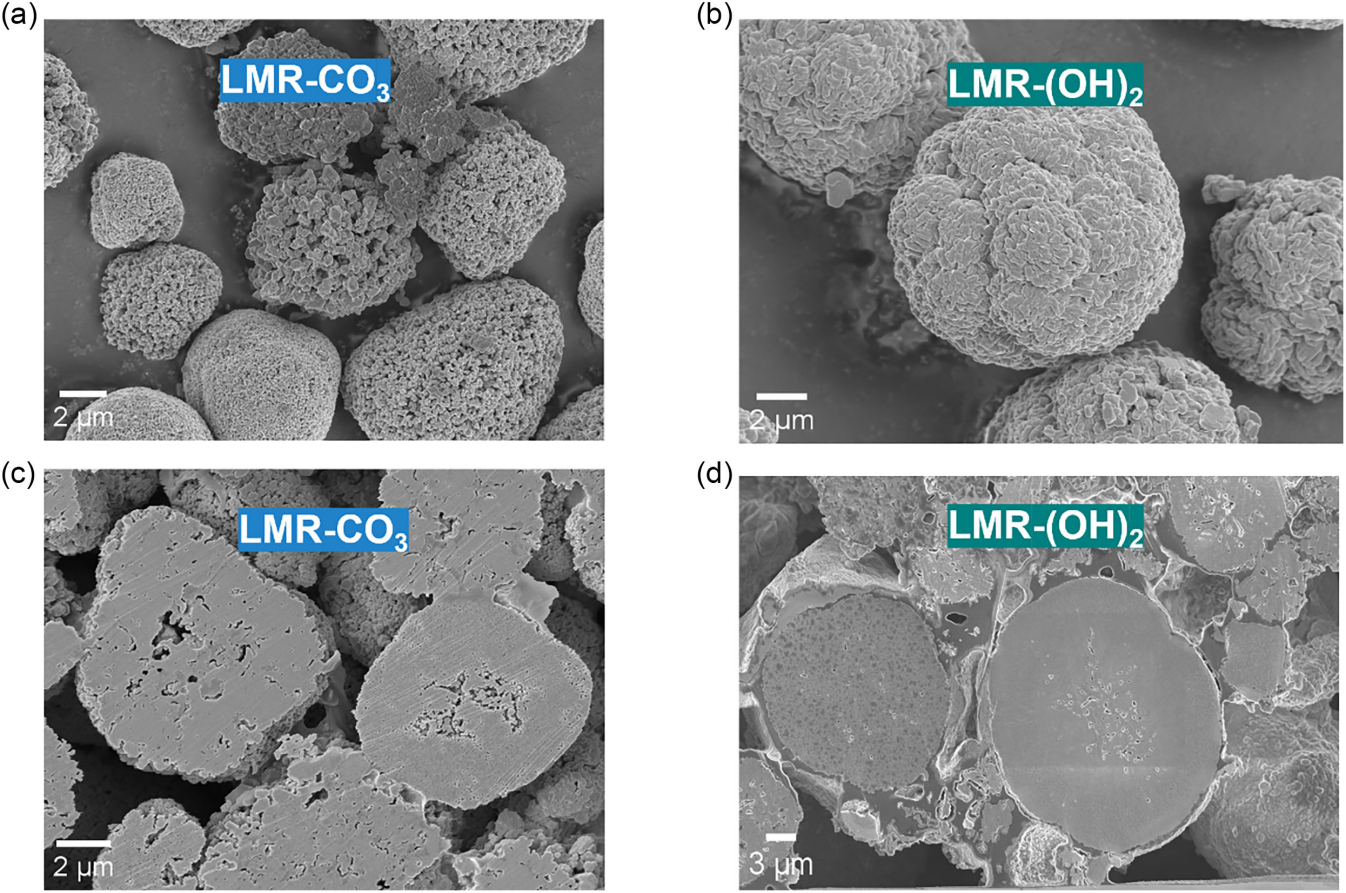
(a) SEM images of LMR‐CO_3_ and (b) LMR‐(OH)_2_ with their respective cross‐sectional images in (c) and (d).

As shown by SEM, LMR‐CO_3_ consists of secondary particles with a wide size distribution (Figure 1a). A higher magnification indicates large, medium, and small primary particles (colored blue, green, and red in Figure S1). More voids are seen within the LMR‐CO_3_ secondary particles, as indicated by cross‐section images in Figure 1c (with a broader view in Figure S2), ultimately resulting in a relatively high specific surface area of 3.76 m^2^ g^−1^ as determined by physisorption measurements based on the BET method. In contrast, the LMR‐(OH)_2_ consists of more tightly packed primary particles (Figure 1b,d), resulting in a specific surface area of 0.72 m^2^ g^−1^, which is comparable to conventional polycrystalline NCMs [28, 29]. The commercially obtained LMR‐CO_3_ has a lower particle size of 6.4 µm, whereas the Taylor reactor synthesized LMR‐(OH)_2_ has a particle size of 11.5 and a narrower particle size distribution (Figure S3), which is again comparable to conventional NCMs.

### 3.2 Structural Characterization

**FIGURE 2:**
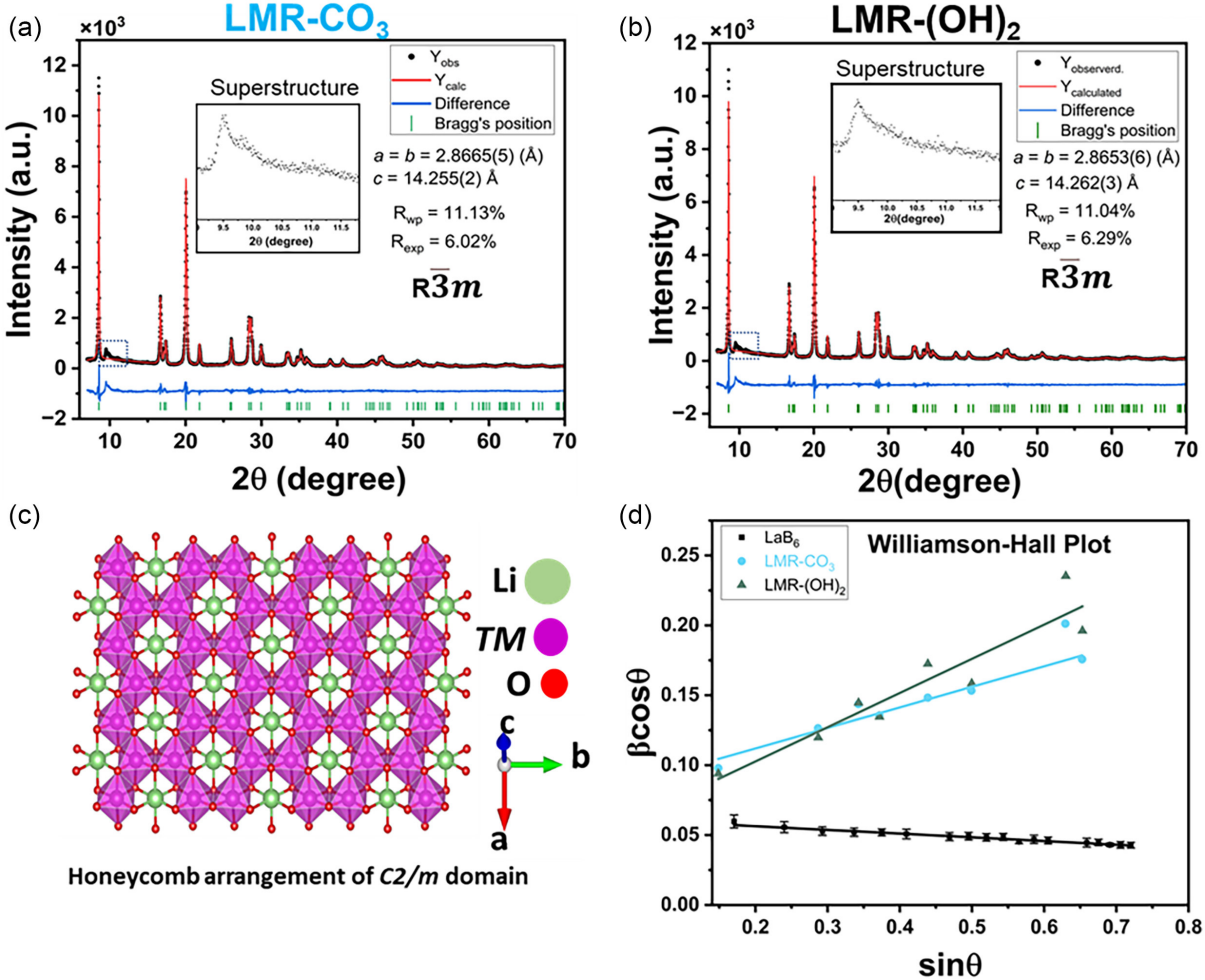
Powder XRD data and Rietveld refinement plot of (a) LMR‐CO_3_ and (b) LMR‐(OH)_2_; within a 0.5 mm capillary using a Mo K_α_‐X‐ray source. Zero shift is adjusted with a LaB_6_ standard. (c) Typical honeycomb arrangement of monoclinic domain. (d) Williamson–Hall plot to distinguish stress and strain.

In a layered oxide‐based LIB cathode, octahedral units of LiO_6_ and TMO_6_ are arranged in alternating layers in a rhombohedral *α*;‐NaFeO_2_ structure (space group $R\overset{―}{3}m$). Nevertheless, in LMR, up to 1/3 of the TMs can be replaced by Li^+^‐ions at the 3a site, which reduces the symmetry and increases the amount of the monoclinic structure (space group *C2/m*). Li in TM layers gives rise to a honeycomb arrangement (Figure 2c), which is characterized by weak and broad reflections at low diffraction angles (2*θ* = 20°–25°) (Figure 2a,b, inset), generally known as superstructure. Insets of Figure 2a,b show the superstructure reflections of both the LMRs showing similar reflection shape and intensity.

The applied Rietveld refinement of the XRD pattern assumes a single‐phase rhombohedral structure with a space group $R\overset{―}{3}m$, with some Li at the TM site for simplicity reasons in order to avoid confusion with a debate about single and multiple phases. Structurally, both LMRs can be regarded as similar, and the refinement data of both pristine LMRs are summarized in Table S2. Apart from the slight line broadening and the intensity increase of the (101), (104), and (003) reflections (shown in Figure S4), no major differences can be observed in the superstructure diffraction pattern (inset in Figure 2a,b).

The line broadening, that is, full width at half maxima (FWHM) is analyzed according to the Williamson–Hall method, where the reflection width (FWHM, *β*) is convoluted into crystallite size and lattice strain according to the equations below (with *K* = Scherrer constant, 0.89, *λ *= wavelength of the X‐ray source, *d* = crystallite size, and *ε *= lattice strain) [30]:

(1) $$\beta_{\text{sample}}=\beta_{\text{crystallite size}}+\beta_{\text{lattice strain}}$$

(2) $$\beta_{\text{sample}}\text{cos}\;\theta =\frac{K\lambda }{d}+4\epsilon \;\sin\;\theta$$

The LMRs are compared with an ideal isotropic broadening material, here standard LaB_6_ (black squares in Figure 2d), which has negligible microstrain, as seen by an almost zero slope. The higher slope for LMR‐(OH)_2_ in the Williamson–Hall plot in Figure 2d points to higher lattice strain compared to LMR‐CO_3._

**FIGURE 3:**
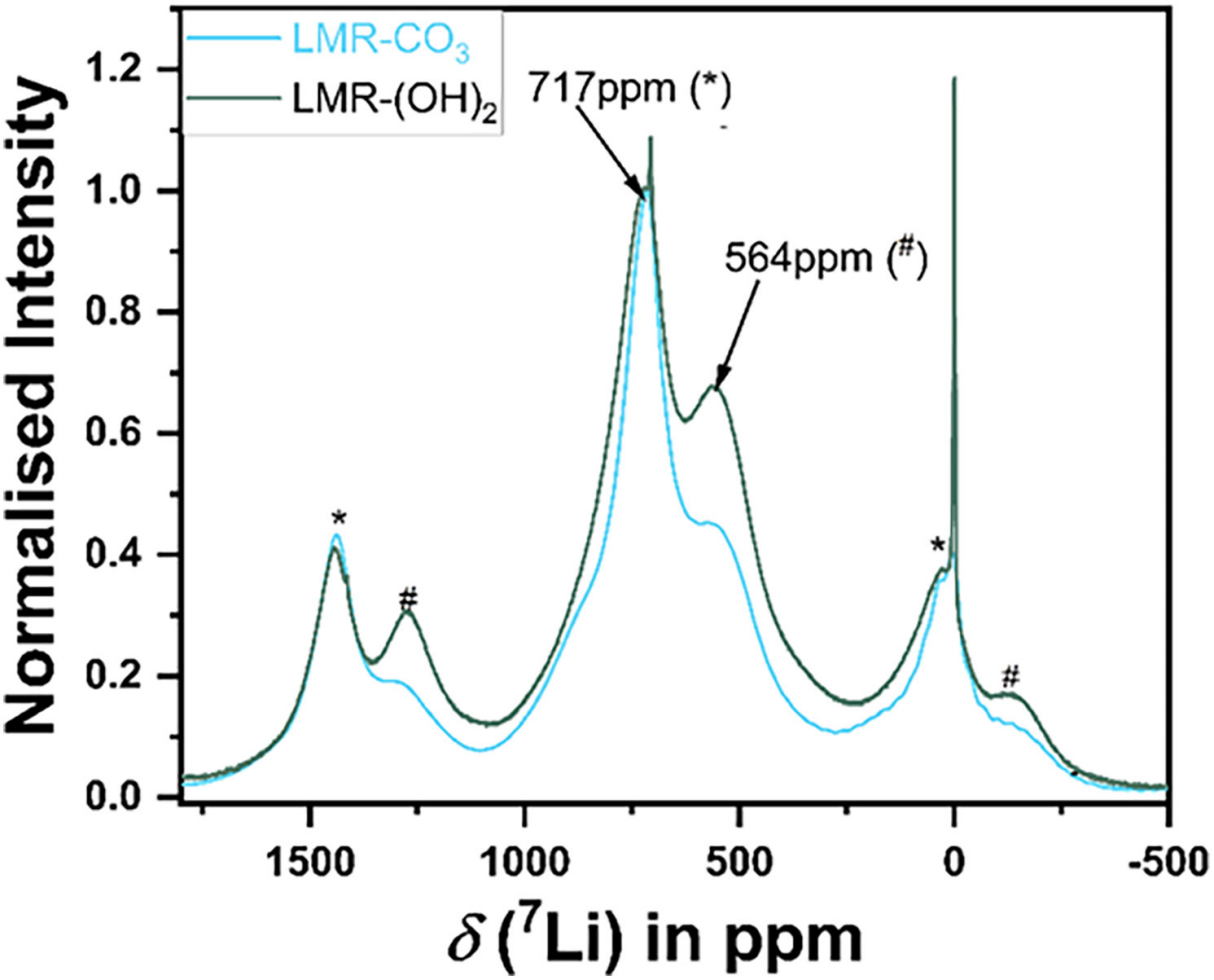
^7^Li MAS NMR spectra of LMR‐CO_3_ and LMR‐(OH)_2_ cathodes, where the intensities are normalized to the resonances at 717 ppm. The higher peak of LMR‐(OH)_2_ at 564 ppm suggests different coordination of Li, likely in course of structural disorder. Spinning sidebands are marked by * and #.

The local structure of Li ions within the positive electrode is unraveled in more detail by ^7^Li MAS NMR spectroscopy (^7^Li‐MAS NMR). ^7^Li spectra of both LMR in their pristine states are shown in Figure 3, where two ^7^Li signals in the chemical shift range between 500 and 800 ppm, that is, at 717 and 564 ppm, are seen. These chemical shifts are in agreement with a recent study of Liu et al. for similar LMR cathodes [31]. Bréger et al. observed two distinct ^7^Li signals at 775 and 759 ppm in monocrystalline Li_2_MnO_3_–Li[Ni_1/2_Mn_1/2_]O_2_ and assigned the resonances to the Li^+^ ions in Li layers (Li_Li_‐_layer_) in the structure [32]. Meanwhile, with increased disorder, but still high crystallinity, multiple signals between 744 and 803 ppm are observed by the authors [32]. These resonances are allocated to Li_Li_‐_layer_, but the increased number of resonances and their chemical shifts can reasonably be attributed to stacking faults and the intergrowth of different structures. Additionally, Grey et al. [33] assigned the resonance 500–600 ppm to Li^+^‐ions in a tetrahedral environment in a spinel structure containing Li─O─Mn bond angle approximately 122°. Furthermore, Bréger et al. showed that stacking faults in Li_2_MnO_3_ produce small changes in Li─O─Mn bond angles, generating an extra ^7^Li NMR resonance [32]. Even slight stacking dislocations create new Li environments with shifted resonances. Additionally, when Ni is incorporated into the TM layer, the Li_Li‐layer_ peak broadens and exhibits a shoulder at around 870 and 546 ppm, respectively, indicating heterogeneity of Ni and Mn coordination to Li [34].

Interestingly, while both LMR cathodes exhibit similar chemical shifts for the main resonances, their relative intensity distributions differ. For LMR‐(OH)_2_, the resonance intensity at 564 ppm is higher relative to the peak at 717 ppm, suggesting a higher population of Li‐ion sites in Li_Li‐layer_. Yang et al. found that this peak at 564 ppm evolves with SoC. They found that during charging (Ni^2+^→Ni^4+^ and Li^+^ removal), the broad Li_Li‐layer_ resonance shifted from 615 down to 555 ppm [34]. They attributed this shift to Ni oxidation and possible Li diffusion from octahedral into tetrahedral sites.

In Ni─Mn oxides, the extra Li and Li/Ni disorder leads to an elongated *c‐*axis and broad diffraction peaks. Sigel et al. observed a strong anisotropic strain along the *c*‐axis in Li─Ni─Mn layered cathodes after overlithiation [34], alongside the peak broadening and increase in *c*‐lattice parameter as characteristic indicators [35]. In summary, the ~564 ppm NMR peak in LMR is associated with Li_Li‐layer_ in regions of high Ni content or disorder. It likely indicates Li in a distorted coordination with varying numbers of Ni^2+^, Mn^4+^, and Li^+^ in the first‐ and second‐cation coordination shells of Li_Li‐layer,_ resulting from Li/Ni mixing and stacking faults [32], consistent with the observed *c*‐axis elongation in LMR‐(OH)_2_ and more pronounced anisotropic lattice strain as also supported by the Williamson–Hall analysis shown in Figure 2d.

To further quantify the distribution of Li environments, spectral deconvolution of the ^7^Li‐MAS NMR spectra is performed for both LMR‐CO_3_ and LMR‐(OH)_2_ (Figure S13a,b). The deconvoluted spectra reveal a consistent trend. All three principal resonances shift toward lower ppm values in LMR‐(OH)_2_ compared to LMR‐CO_3_. For instance, the resonance exhibiting the strongest Fermi contact shift appears at 864 ppm in LMR‐CO_3_, while in LMR‐(OH)_2,_ the most downfield‐shifted peak is located at 790 ppm, which can also be related to enhanced lattice strain. However, due to the limitations of the acquired spectra, definitive conclusions regarding the presence and structural characteristics of Li^+^‐ions in TM‐layers (Li_TM‐layer_) remain elusive and warrant further investigation. Resonances at approx. 1500 ppm [32] would correspond to Li_TM‐layer_ due to an even more pronounced Fermi contact shift. But those resonances are interfered with by rotational sidebands from ^7^Li resonances at 564 and 717 ppm, marked with (*) and (#) in Figure 3. To unravel the local structure of Li_TM‐layer_ further experiments such as ^6^Li NMR spectra or ^7^Li pjMATPASS spectra [36] are required, but are beyond the scope of this study due to the low natural abundance of ^6^Li and limitations in the magnetic field (4.7 T).

### 3.3 Charge/Discharge Cycling

**FIGURE 4:**
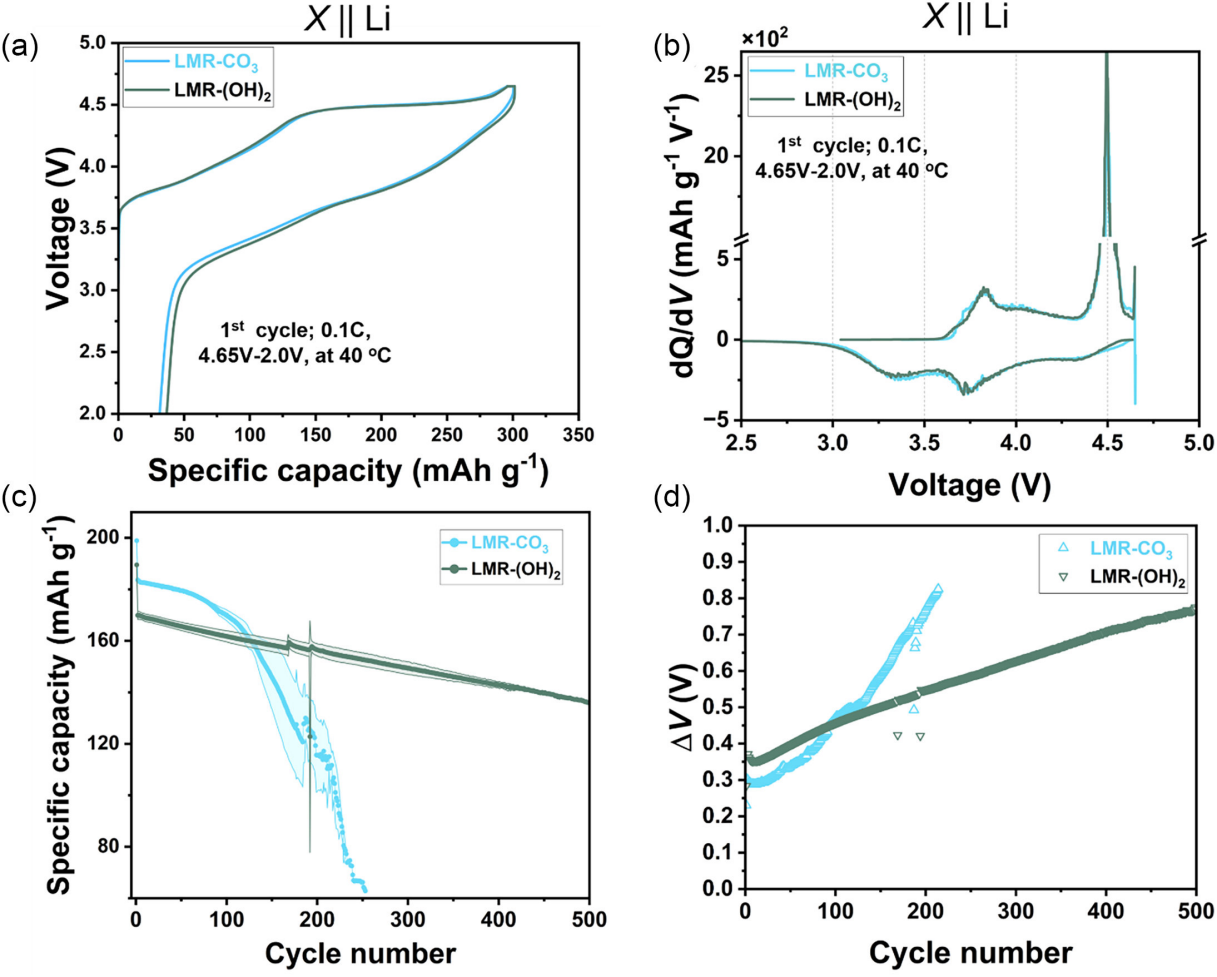
(a) First cycle voltage profiles of LMR‐CO_3_ and LMR‐(OH)_2_ coin cells at 0.1 C within the voltage range of 4.65–2.0 V at 40 °C (1 C ≙ 180 mA g^−1^), and (b) their corresponding *d* *Q*/*d* *V* versus voltage plots. (c) Initial 500 cycles and (d) average voltage versus cycle number indicating an increase in cell resistance.

**FIGURE 5:**
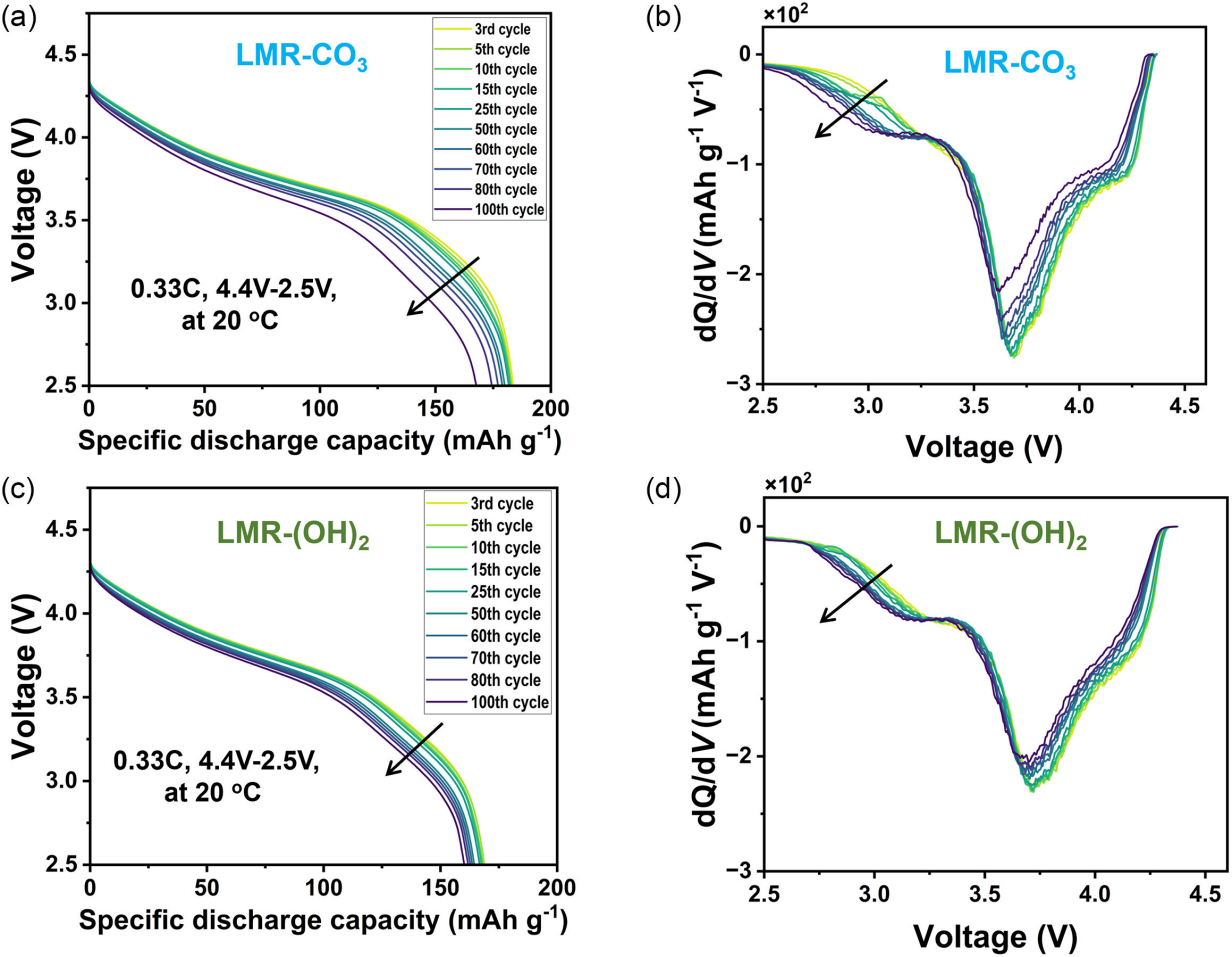
Discharge profiles of (a) LMR‐CO_3_ and (c) LMR‐(OH)_2_ of 3rd to 100th cycle and their corresponding *d* *Q*/*d* *V* versus voltage plots in (b) and (d) respectively, indicating degradation in the lower voltage region.

As shown in Figure 4a, both materials provide similar specific charge capacities in Li‐metal cells, but LMR‐(OH)_2_ has a lower specific discharge capacity (Figure S5). The first cycle is charged/discharged at 40 °C in order to obtain a practically high delithiation capacity, that is, stemming from oxide‐redox and is literature‐known as “activation” [10] and would be ~30 mAh g^−1^ lower at 20 °C (Figure S16). Nevertheless, LMR‐(OH)_2_ shows better cycle life (capacity retention) in the voltage range of 4.4–2.5 V at 0.33 C (Figure 4c), where 1 C ≙ 180 mA g^−1^. The evolution of the discharge voltage profiles up to 100^th^ cycle (Figure 5a–d) indicates higher fade at a lower voltage region (3.5–3.0 V) for both materials, whereas LMR‐CO_3_ degrades more. Voltage hysteresis, being e.g., an indicator for cell resistance [37, 38], determined by difference between average charge and discharge voltage (∆*V*) in Figure 4d is initially lower for LMR‐CO_3_ compared to LMR‐(OH)_2_, which however increases and is higher after ~100 cycles. The *d* *Q*/*d* *V* versus voltage plots of synthesized LMR‐(OH)_2_ CAM (Figure 4b) mainly differ from LMR‐CO_3_ in the lower voltage region of 3.6–3.0 V during discharge, which can be attributed to Mn^3+^/Mn^4+^ redox [31, 39]. According to Merz et al., this Mn redox takes place on the surface [39], thus correlating with the higher specific surface area of LMR‐CO_3_ and likely leading to a different degree of oxygen evolution [40].

The rate performance analysis is shown in Figure S6, where a difference of ~20 mAh g^−1^ discharge capacity is seen at C‐rates < 1 C. At higher C‐rates, LMR‐(OH)_2_ provides higher capacity and shows better performance than LMR‐CO_3_.

**FIGURE 6:**
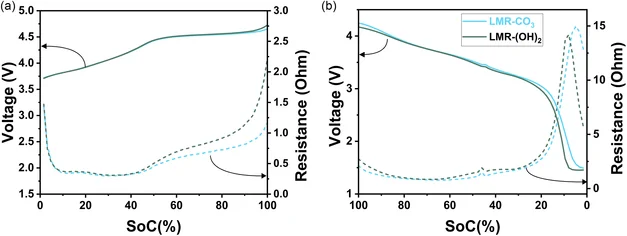
Voltage and resistance curves as a function of SoC during first cycle (a) charge and (b) discharge. Resistance differences are observed for oxygen redox during charge only and can be related to the higher lattice strain of LMR‐(OH)_2_.

Higher fading in specific capacity of LMR‐CO_3_ correlates with increasing voltage hysteresis (Figure 4d). The SoC‐dependent resistance during the first formation cycle is evaluated via GITT (Figure 6a,b, more details in Figure S7). During charge (Figure 6a), the resistance decreases rapidly at low SoC (<10%) and remains at ~2 Ω until 40% SoC. This SoC dependence is typical for TM redox [31, 39]. The plateau above 4.3 V can be assigned to anionic redox [8, 31], which is accompanied by resistance rise for both LMRs, whereas the increase for LMR‐(OH)_2_ is slightly higher, while during discharge, the resistances are similar again (Figure 6b), except the end of discharge, suggesting differences in redox reaction resistivity, that is, less resistance for Mn^4+/3+^ redox, with pronounced higher resistance difference than during charge, and can be also correlated with higher lattice strain of LMR‐(OH)_2_ observed from XRD and ^7^Li NMR data.

In addition to intrinsic structural factors, extrinsic electrode‐level parameters need to be considered when comparing the performance of LMR‐CO_3_ and LMR‐(OH)_2_. In addition to particle size distribution, the hollow, porous secondary‐particle morphology of the carbonate‐derived material decreases tap density, which reduces volumetric energy density. With lower‐density electrodes, the thickness of cathode coating is higher for the same areal capacity. This can result in an altered cathode composition, leading to changes in ionic/electronic resistances and indirectly decreasing gravimetric, that is, specific energy. Therefore, electrochemical differences observed between the two materials can also stem from these electrode‐level constraints [41, 42].

### 3.4 LMR‐(OH)_2_ Calcination: Structural Evolution via In Situ XRD and TGA

**FIGURE 7:**
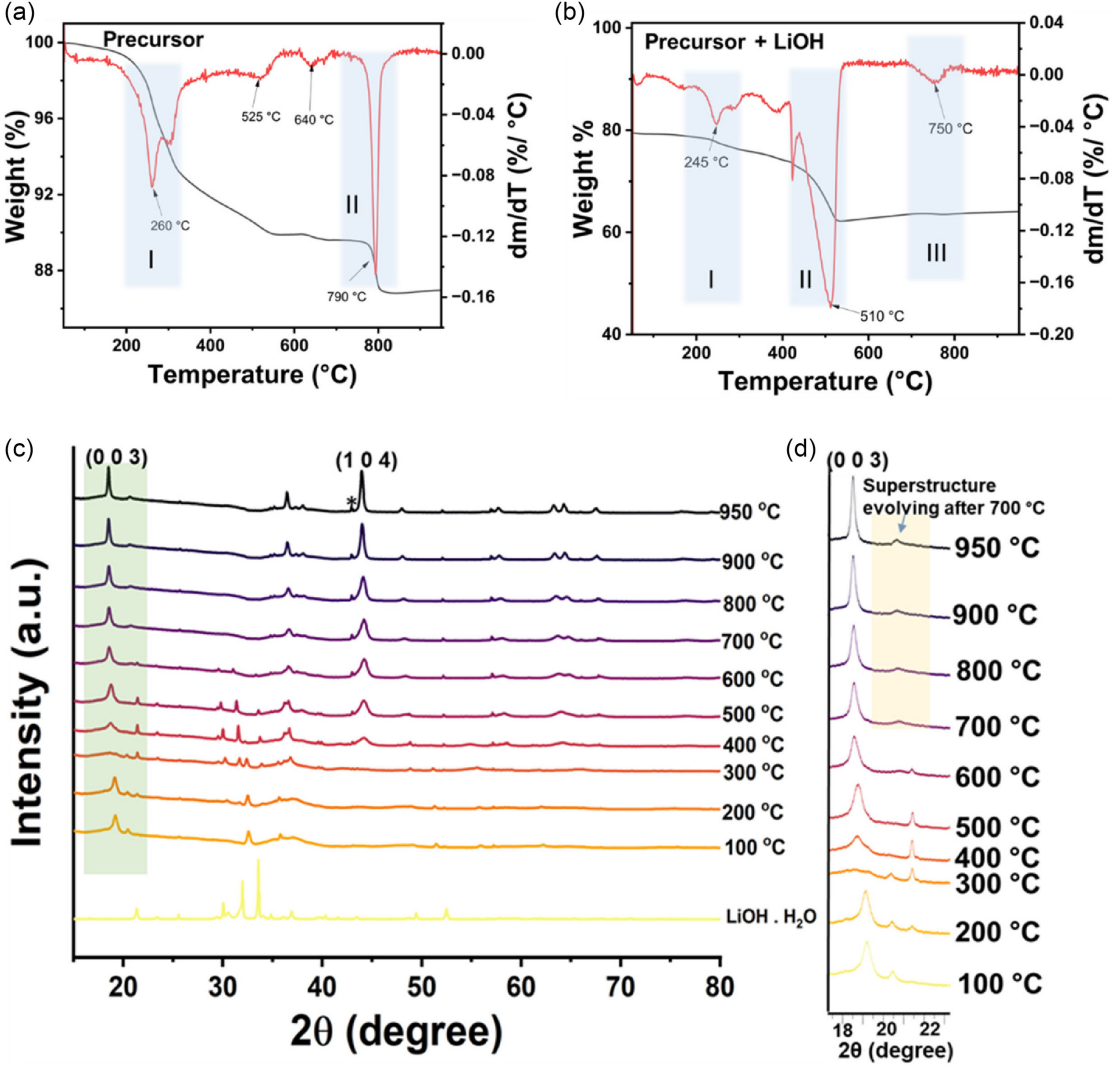
TGA curve of (a) hydroxide precursor and (b) hydroxide precursor/LiOH mixture within the temperature range of 50 to 950 °C. (c) In situ XRD data of only LiOH·H_2_O (yellow line) and hydroxide precursor/LiOH at temperatures from 100 to 950 °C. (d) Zoomed (003) reflection and evolution of superstructure around 20° above 700 °C.

The TGA profiles of the hydroxide precursor as well as the mixture of the hydroxide precursor with LiOH (Li source for calcination) are shown in Figure 7a,b, respectively. In the precursor (Figure 7a), the initial weight loss of ~3% below 200 °C can be attributed to the removal of adsorbed water, while the second weight loss of ~7% in (region “I”, Figure 7a) is due to the dehydration of the oxy‐hydroxy precursor TM‐OOH. The third weight loss at 525, 640 °C, and particularly at 790 °C (region “II,” Figure 7a) can be assigned to final dehydration toward TMO_2_ phases [43].

In contrast, the hydroxide precursor/LiOH mixture (Figure 7b) can be divided into three regions. In region I (<355 °C), ~20% mass loss is related to the dehydration and decomposition of LiOH (to form Li_2_O and H_2_O↑), which correlates with the shrinkage of the LiOH reflections in XRD (Figure 7c). The mass loss in region II (400–550 °C) is related to the Li‐ and TM‐slab separation in the course of Li incorporation into the Li‐slab, oxidation of TMs via oxygen reduction, and release of water to form a final layered oxide LMR. This correlates with the evolution of Bragg’s reflections (003) and (104) in Figure 7c.

As LiOH melts at 462 °C, the lithiation into Li‐slab occurs via a solid–liquid reaction, contrary to solid–solid reaction with Li_2_CO_3_, which melts at 723 °C [44, 45]. This solid–liquid reaction via the hydroxide precursor and LiOH mixture can correlate with stacking faults or distorted octahedra site occupation of Li_Li_‐_layers_ as previously observed via ^7^Li‐MAS NMR results.

The solid–liquid phase reaction gives rise to a trigonal‐type intermediate, which can be seen by ex situ calcination XRD during the holding time at 500 °C, as shown in Figure S9. This intermediate appears after 1 h of holding at 500 °C and increases in intensity with time. This is a pseudolayered phase, which later turns into a layered phase and gives rise to $R\overset{―}{3}m$ symmetry. Note that the rock‐salt phase (*Fd‐*3*m*) and the layered phase ($R\overset{―}{3}m$) exhibit similar reflection positions at 2θ and cannot be distinguished properly.

Nevertheless, they all morph into a layered rhombohedral structure with space group $R\overset{―}{3}m$ in the course of calcination. Hints for a spinel phase reflection (104) (asterisk * in Figure 7c) are only present in in situ XRD measurements and can be related to excess LiOH within a small sample holder (<1 g). This spinel phase is not present in ex situ calcination (Figures S9, S10).

The TGA curve in Figure 7b shows that the rate of mass loss is not constant throughout the reaction temperature. Region III (690–800 °C, Figure 7b) shows a slow and steady state where Li inserts into the TM layers giving rise to the superstructure. This observation is supported by XRD data in Figure 7d, where the superstructure evolves after 700 °C. This change in lithiation rate may be related to smaller Li^+^ diffusion kinetics into the TM layer [46, 47], where both the Li layer and TM layer are already overwhelmingly occupied and increase the barrier for Li^+^ hopping into the remaining vacant sites [44]. Nevertheless, it can be concluded that solid–liquid phase reaction during lithiation can induce stacking faults, lattice stress, and elongate the *c*‐lattice parameter, which is in line with the data from the Williamson–Hall plot in Figure 2d and the ^7^Li‐MAS NMR study.

### 3.5 Operando XRD During Formation Cycle

**FIGURE 8:**
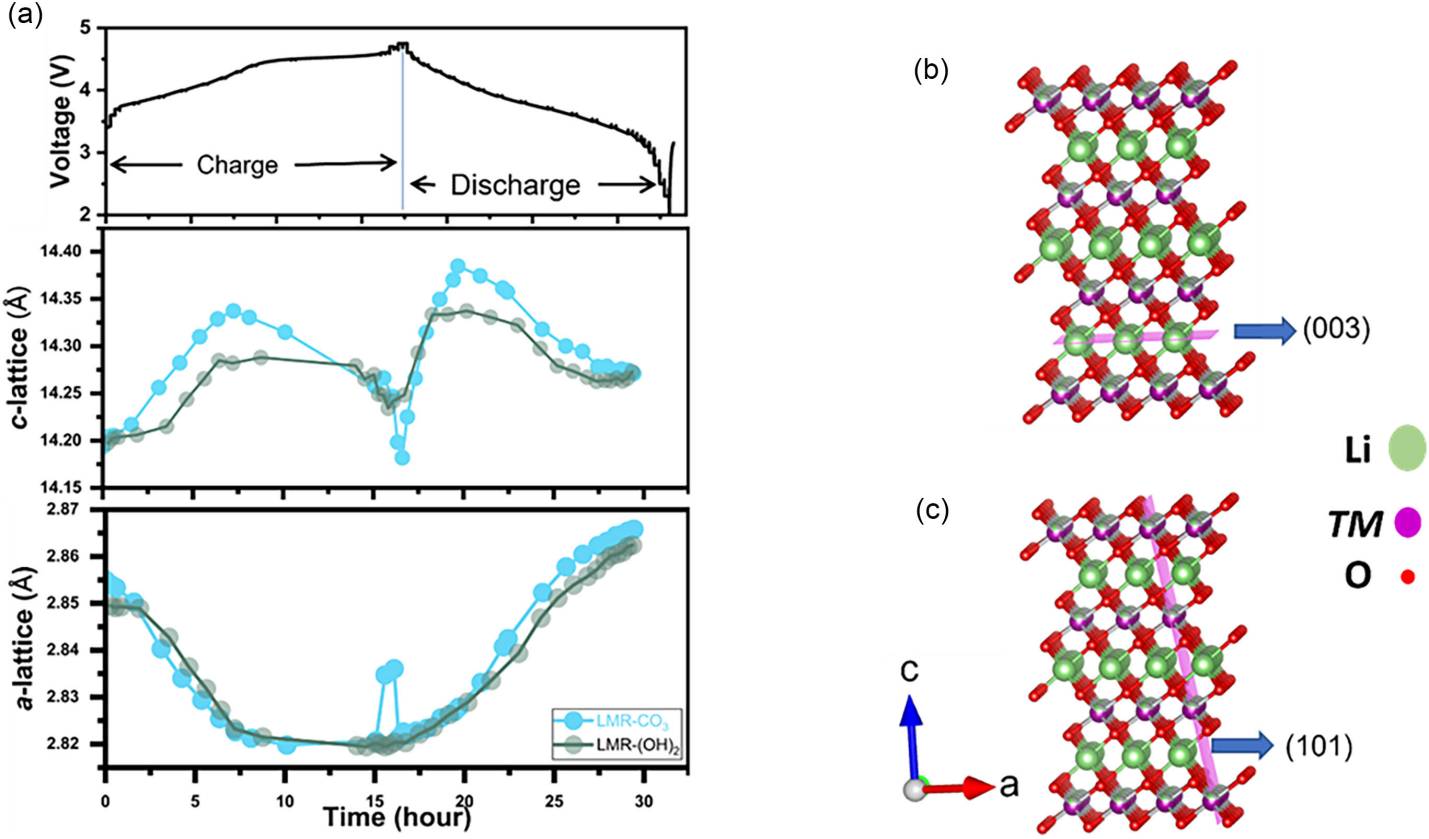
(a) Voltage and (*a*‐, *c*‐) lattice parameter of LMR‐CO_3_ (black) and LMR‐(OH)_2_ (red) as a function of time during the first charge/discharge cycle. The values were obtained by Pawley fitting of the XRD patterns assuming space group $R\overset{―}{3}m$. Visualization of the (b) (003) and (c) (101) lattice planes.

Both LMRs are subjected to operando XRD measurements to investigate the evolution of their crystal structures during the first charge/discharge cycle. The operando XRD patterns of both LMRs (Figure S14; obtained using a modified AMPIX cell, Figure S15; as detailed in the experimental section, cycled at 0.1 C within 4.75–2.0 V at room temperature) show an evolution of the refined lattice parameters *a* and *c*, as presented in Figure 8, overwhelmingly aligning with trends previously reported in the literature [48, 49]. During the initial stages of charging, the *c*‐lattice parameter of the LMR cathode material gradually increases, indicative of an expansion in the interlayer spacing. In parallel, the *a*‐lattice parameter decreases, which can be attributed to a distance shortening due to enhanced TM‐hardness and TM–O covalent hybridization in the course of TM oxidation.

During LMR‐CO_3_ delithiation, the number of vacant sites in the TM layer increases, which enlarges the electrostatic repulsion of the surrounding oxide anions and counteracts the aforementioned shrinkage in the *a*‐lattice parameter. This trend has been observed for low‐nickel NCMs, as reported by Manthiram et al. under deep charge conditions [50]. While both compositions possess comparable total lithium content, stoichiometry, and specific charge capacities, the relithiation during subsequent discharge is lower for LMR‐(OH)_2_. While the *c*‐parameter is similar for both LMR materials, the lower *a*‐parameter suggests a lower volume of the crystal, that is, incomplete discharge, either due to volume change‐resistive redox at end‐of‐discharge (suppressing Li^+^ intercalation) and/or resistive Li^+^ intercalation (suppressing the redox), as schematically shown in Figure 12.

### 3.6 Surface Species Before and After Cycling

**FIGURE 9:**
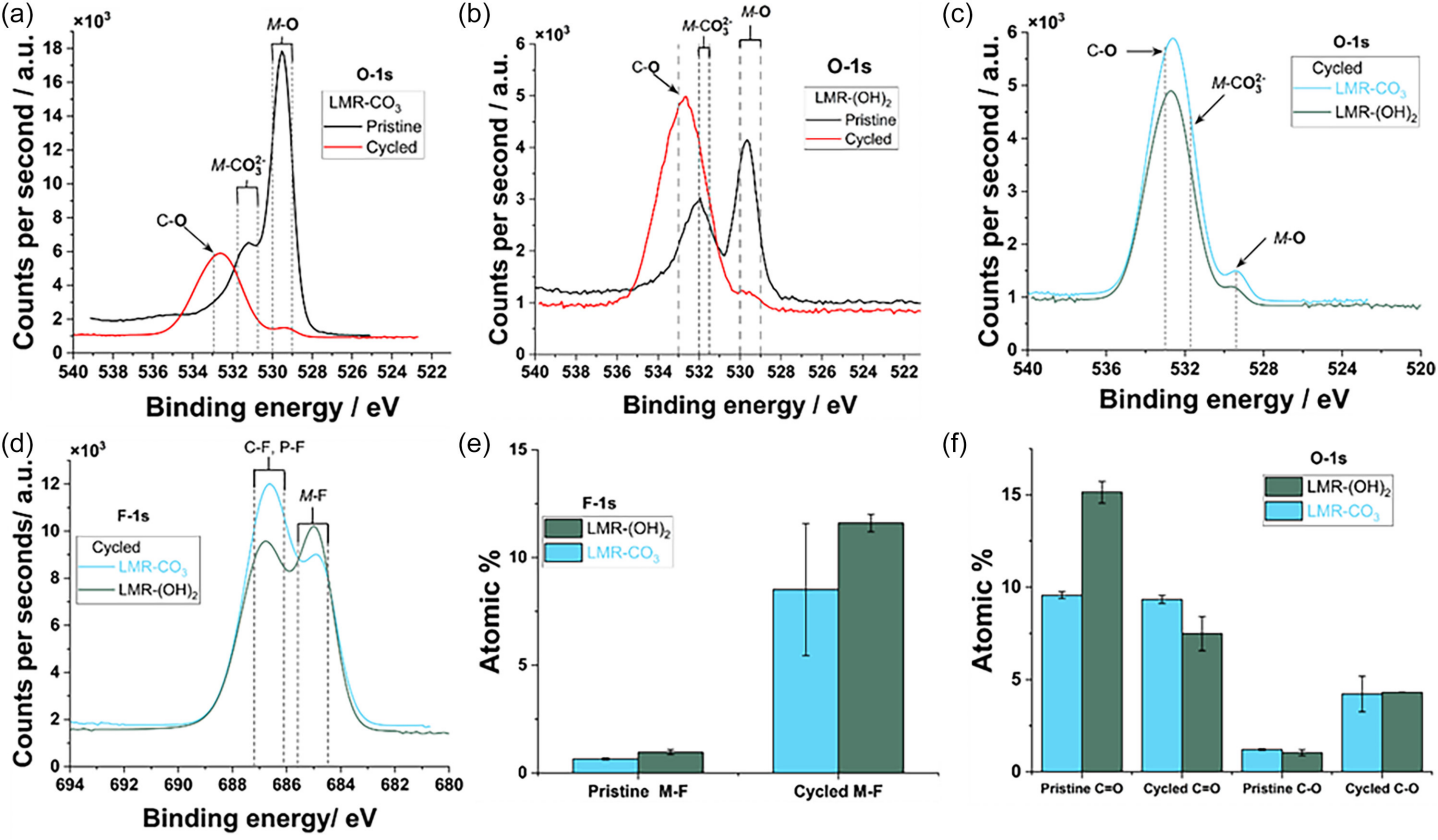
XPS spectra of the O‐1s region of pristine and cycled electrodes of (a) LMR‐CO_3_, (b) LMR‐(OH)_2_ and (c) overlay of cycled electrodes after 100 cycles. (d) F‐1s spectra indicating a higher amount of *M*‐F on LMR‐(OH)_2_. (e) Surface concentration of the M‐F component in pristine and cycled electrodes of both LMRs, indicating an increase in *M*‐F concentration over the course of cycling, which can, among others, stem from reactions of LiPF_6_ with surface Li_2_CO_3._ (f) Surface concentration of C─O‐and C═O components in pristine and cycled electrodes.

Surfaces of the pristine and cycled materials are investigated by Warder titration and XPS. Warder titration is used to determine the amount of hydroxide‐ and carbonate‐containing species [26]. The titration curves and mass concentrations of surface ─OH^‐^ and ─CO_3_^2−^ contents for both LMRs are given in Figure S8 and Table S3. Interestingly, LMR‐(OH)_2_ reveals more surface species and even contains more ─CO_3_^2−^ (─OH^−^ 0.23 wt% and CO_3_^2−^ 0.23 wt%) compared to LMR‐CO_3_ (0.13 wt% ─OH^‐^ and 0.16 wt% CO_3_^2−^). The unreacted LiOH during calcination, which was initially provided in excess amounts (LiOH/TM = 1.35 molar ratio), is suggested to convert into Li_2_CO_3_ with CO_2_ contact. This is also evident from XPS data in Figure 9a,b,f, where the relative intensity of the *M*‐CO_3_ component in the O‐1s spectra of the pristine sample at around 532 eV is higher in LMR‐(OH)_2_ than in LMR‐CO_3_. Postmortem‐harvested LMR cathodes after 100 cycles are processed in an inert atmosphere for XPS investigation (opened in a glovebox and transferred through an airtight box). The M‐CO_3_ component peak of the O‐1s spectra of LMR‐(OH)_2_ samples is less intense compared to pristine and is similar to LMR‐CO_3_ (Figure 9c,f). In contrast, the *M*‐F components' peak intensity in the F‐1s spectra (Figure 9d,e) in the case of the LMR‐(OH)_2_ samples remains higher. Figures S11 and S12 contain XPS spectra of all the carbon‐containing species and their spectra along with the survey scan. It can be concluded that the Li_2_CO_3_ surface species decompose to CO_2_ and Li–F. A thicker Li–F layer is speculated to be beneficial and can additionally prolong the cycle life of LMR‐(OH)_2_ [51].

### 3.7 Discussion

The difference in discharge capacity and cycle life of the investigated LMRs can be related to bulk structural differences, in addition to particle morphology and surface area. Structural differences are seen in slight broadening and positional shifts of the (003), (101), and (104) reflections in XRD patterns for LMR‐(OH)_2_. ^7^Li MAS NMR combined with in situ high‐temperature XRD during calcination hints at stacking faults between TM‐O_6_ and LiO_6_ slabs in the LMR‐(OH)_2_. These differences may stem from the heterogeneous nature of the solid–liquid lithiation reaction at elevated temperatures. Other than Li_2_CO_3_ as a lithiation agent during calcination for LMR‐CO_3_, LiOH melts before CAM lithiation. Differences in the crystal structure can also be reflected in the oxygen redox during charge, in particular during discharge, either via GITT, which indicates higher resistances for LMR‐(OH)_2_ during anionic redox, or via operando XRD indicating less volume expansion due to a lower *a*‐parameter increase in LMR‐(OH)_2_ at end‐of‐discharge for LMR‐CO_3_.

**FIGURE 10:**
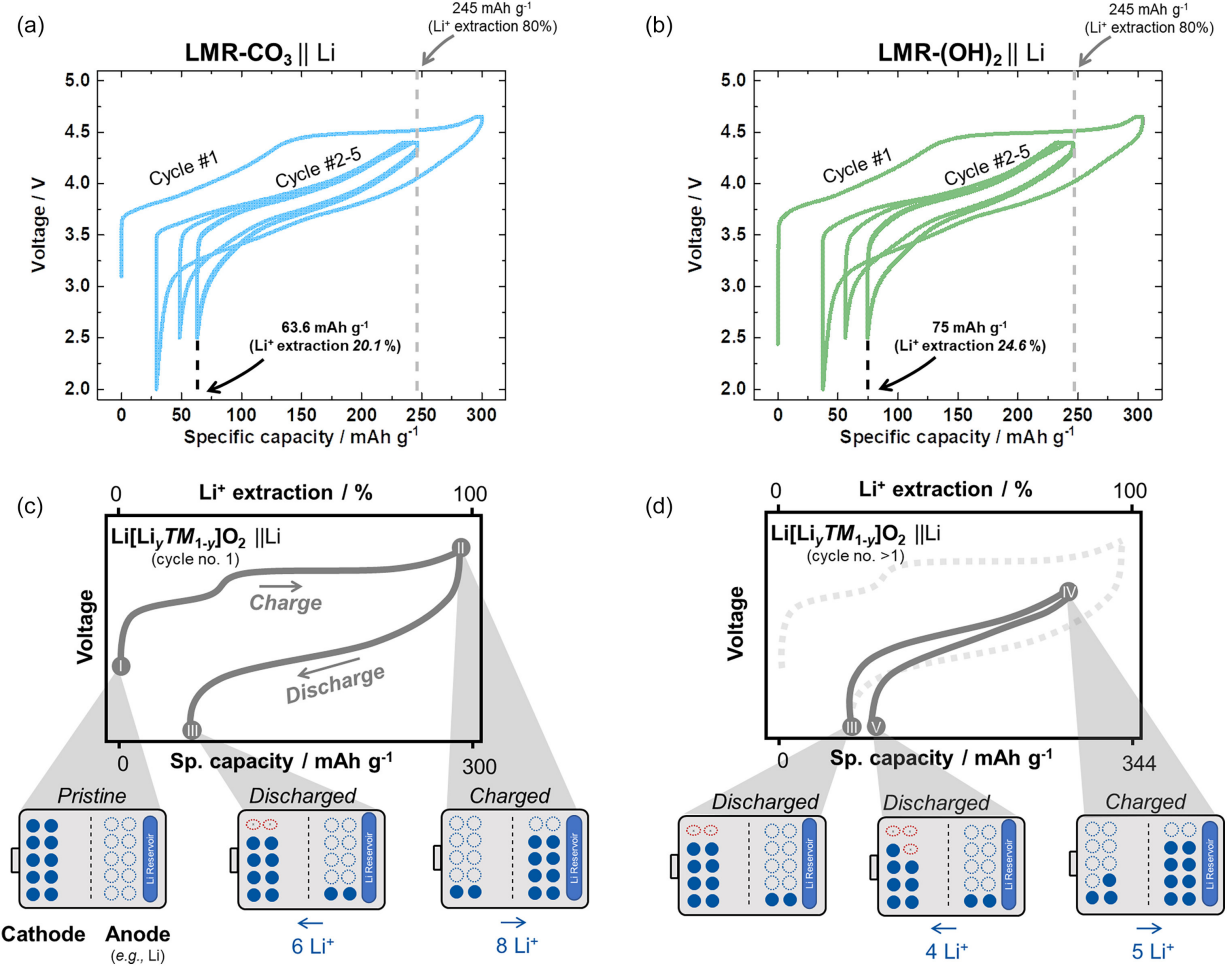
The voltage versus specific capacity plots of the initial five charge/discharge cycles of (a) LMR‐CO_3_ and (b) LMR‐(OH)_2_. Here, the first cycle is operated with 0.1 C at 40 °C within 4.65–2.0 V for reasons of “activation,” that is, to maximize the capacity in particular stemming from O^2‐/0^ redox. Afterward, the cells are operated at 0.33 C at 20 °C within 4.4–2.5 V. While the Li^+^ extraction during charge is similar, the lithiation amount during discharge differs. Incomplete lithiation suggests a different Li^+^ extraction ratio in the discharged state, as schematically shown for the first (c) and subsequent cycles (d). Given the lower lithiation amount of LMR‐(OH)_2_, thus the higher Li^+^ extraction in the discharged state, a higher oxidation number of the redox can be concluded; consequently, a lower amount of detrimental Mn^3+^.

Differences in cycle life cannot be caused by differences in initial Li^+^ extraction ratio (similar SoC) [29] as the initial specific charge capacities are similar following the assumption that no parasitic reaction takes place [52, 54]. Instead, the higher capacity retention of LMR‐(OH)_2_ can be attributed to the combination of several aspects: lower surface area compared to LMR‐CO_3_, which has less irreversible oxygen evolution and intertwined detrimental Mn^3+/4+^ redox activity. Though in the latter the capacity theoretically remains similar, as only the redox activity is shifted from oxides to Mn, the detrimental Mn^3+^ is also known to cause and trigger structural instabilities, while initially—apparently beneficially—enabling more lithiation capacity at end‐of‐discharge (Figure 10) [55]. An increased amount of Li–F on the surface may further contribute positively to the cycle life, as well as different particle size distribution.

## 4 Summary and Conclusion

**FIGURE 11:**
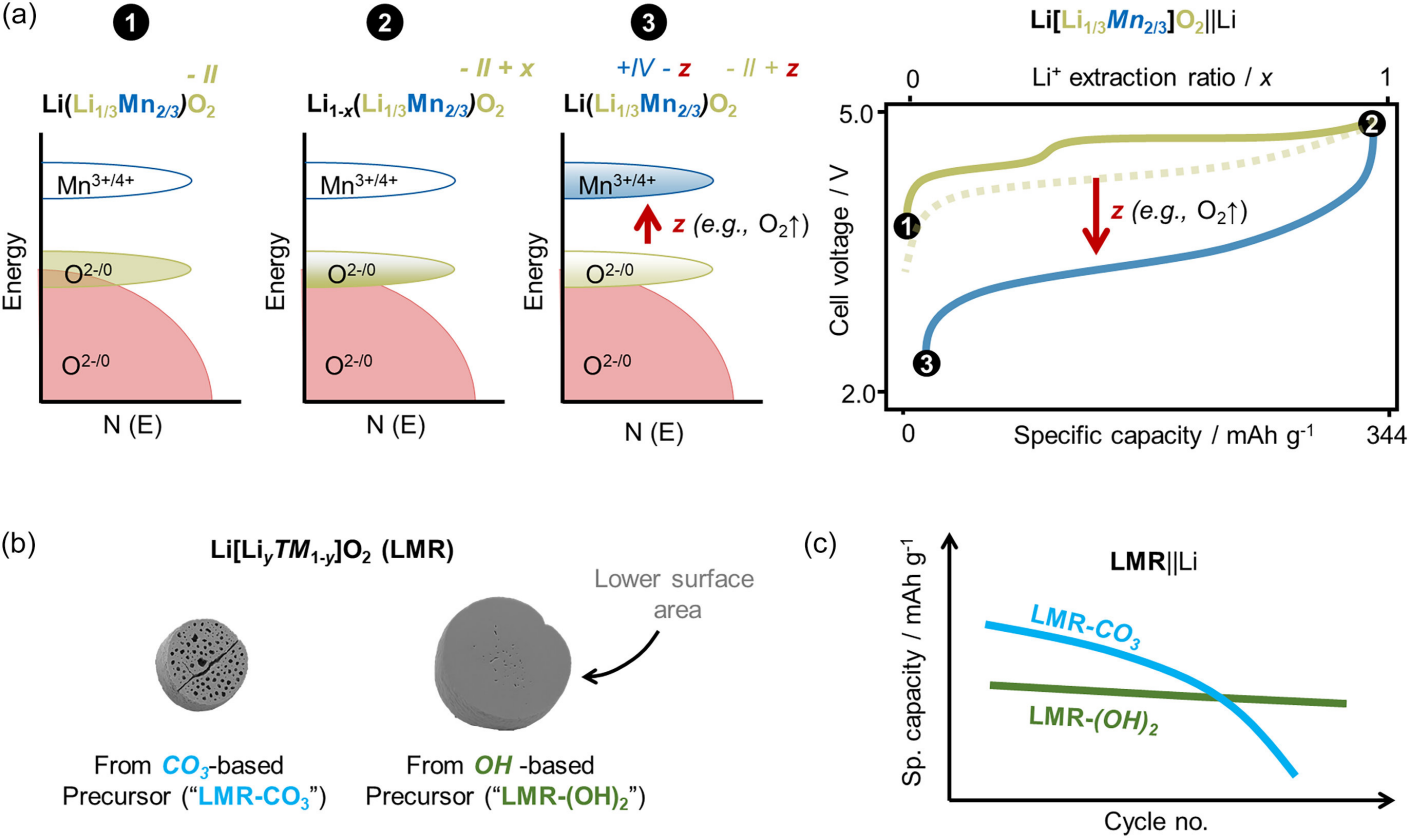
(a) In LMR materials, the capacity is aimed to stem from O^2−/0^ redox. In practice, however, this redox activity can decrease (e.g., due to O_2_ evolution) and shifts the redox toward Mn^3+/4^, which not only decreases discharge voltage (“voltage fade”) but also can destabilize the structure due to Mn^3+^‐reasoned Jahn–Teller distortions and disproportionation reactions. (b) Schematic representation of LMR‐CO_3_ morphology with internal voids and hence higher surface area compared to LMR‐(OH)_2_. (c) Schematic representation of long‐term cycle life of the LMRs. Though LMR‐(OH)_2_ has a lower lithiation amount, thus less capacity, it is beneficial for the cycle life, as the lower lithiation amount is accompanied by less detrimental Mn^3+^.

Layered oxide‐based cathodes, such as LiTMO_2_ (TM = Ni, Co, Mn), are SOTA materials due to their high specific capacity, mean discharge potential, and good electrical conduction (e^−^ and Li^+^), consequently possessing high specific energy and power. A measure to further increase the specific energy is to partly exchange TM with Li in the TM layer, yielding Li[Li*_y_* TM_1−*y*_]O_2_ (1 < *y* < 1/3). Given the respective rise in TM oxidation state toward +4, a high Mn amount is possible, as the Mn^4+^ is known to be beneficial in terms of thermal stability, sustainability, and relatively to other TMs—costs and molar mass. These so‐called Li/Mn‐rich materials (LMR) have a higher specific capacity due to lower molar mass and a higher electrode potential due to O^2−/0^ redox. In practice, however, O^2−/0^ redox diminishes due to, for example, irreversible oxygen release, and the redox activity shifts toward Mn^3+/4+^, which decreases mean discharge voltage and structural degradation due to Mn^3+^‐related Jahn–Teller distortion and disproportionation reactions (Figure 11a).

**FIGURE 12:**
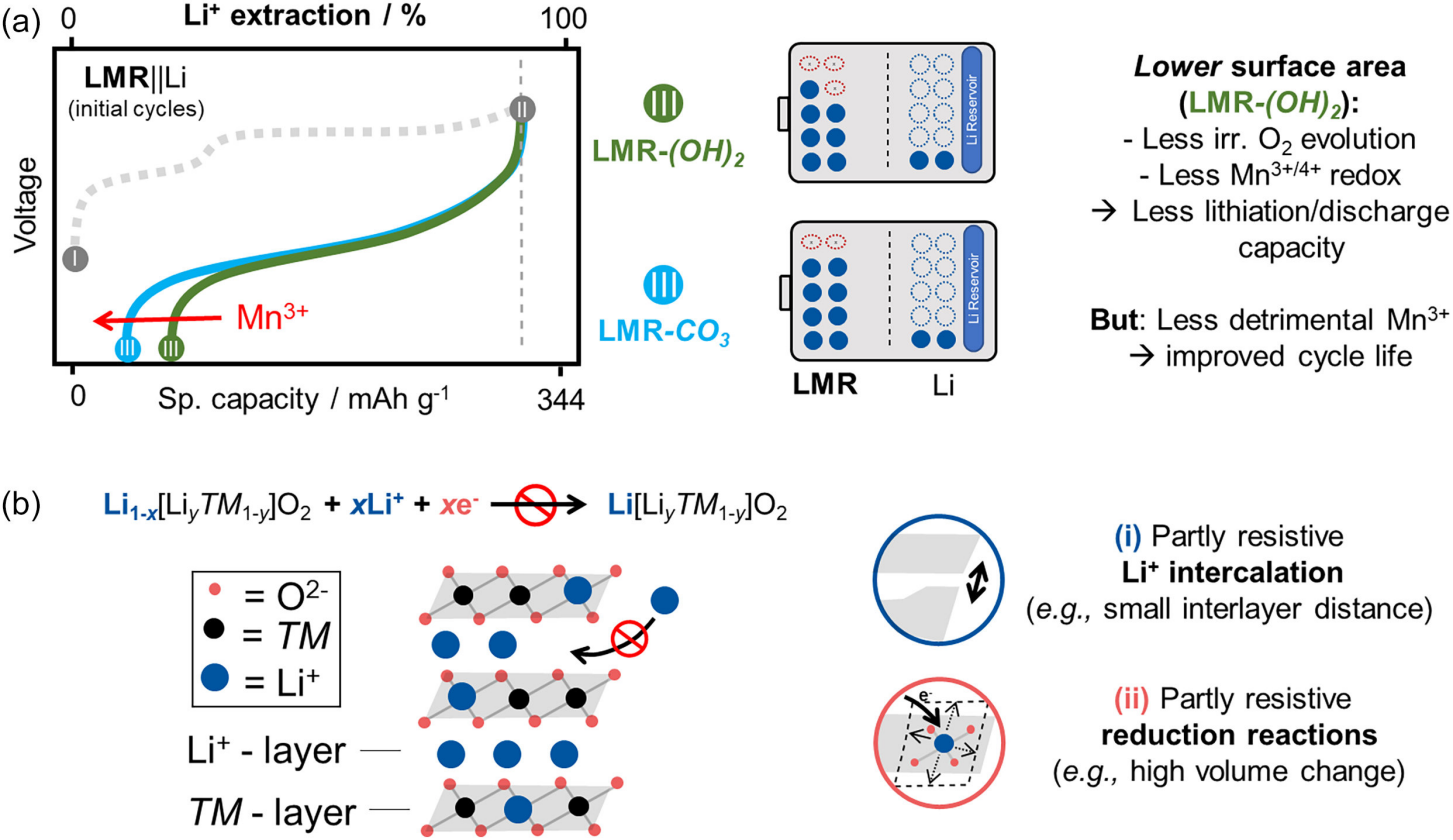
(a) More lithiation capacity is concluded to be detrimental as it requires more Mn^3+/4+^ redox and enhances the amount of Mn^3+^, which is concluded to destabilize the structure due to Jahn–Teller distortion and disproportionation reactions. (b) The reason for the higher lithiation amount of LMR‐CO_3_ can, among others, be related to less resistive volume/phase changes of different redox at end‐of‐discharge conditions according to scenario (ii), or more likely to resistive Li^+^ intercalation according to scenario (i) as hinted by bigger particles and observed (slight) stacking faults.

In this work, the LMR is synthesized via OH^−^ coprecipitation and subsequent LiOH‐based calcination (LMR‐(OH)_2_) and compared with conventional LMR synthesized via coprecipitation with CO_3_^2−^ and calcination with Li_2_CO_3_ (LMR‐CO_3_). In this way, the gas evolution‐related (CO, CO_2_) voids and high surface area are suppressed, and an LMR‐(OH)_2_ with lower surface area (0.72 vs. 3.76 m^2^ g^−1^) is obtained. Interestingly, the specific charge capacity, thus Li^+^ extraction ratio and SOC, is similar for both materials in the initial cycles, while the lithiation in the LMR‐CO_3_ during discharge is higher (~12 mAh g^−1^). However, this (apparently beneficial) increased specific discharge capacity is shown to stem from detrimental Mn^3+^ redox and is finally concluded to be disadvantageous, as it can cause the subsequent severe capacity fade compared to LMR‐(OH)_2,_ as shown in Figure 12a.

Consequently, LMR‐(OH)_2_ can be concluded as beneficial in terms of cycle life due to less Mn^3+/4+^ redox because of less lithiation during discharge. This incomplete lithiation either stems from resistive lithiation (Li^+^ + e^−^) kinetics, due to (i) partly resistive Li^+^ intercalation, for example, lower Li^+^ interlayer distance, or due to (ii) partly resistive redox itself, for example, due to accompanying volume changes (Figure 12b) at end‐of‐discharge and requires more in‐depth investigations.

In this work, evidence for resistive Li^+^ intercalation (scenario i) is observed. In line with conventional LiTMO_2_, smaller particles tend to have lower capacity loss, that is, a higher lithiation degree into the structure, as, for example, known for the smaller primary particles in polycrystals compared to single crystals [56]. Less lithiation during discharge, thus less detrimental Mn^3+/4+^ in LMR‐(OH)_2_, can be attributed to higher particle sizes and/or more lattice strain, as concluded from the Williamson–Hall plot by means of XRD. According to ^7^Li MAS NMR spectroscopy, the lattice strain may also stem from stacking faults, which can affect the Li^+^ intercalation resistances. In fact, differences in resistances are observed via the GITT particularly at the end‐of‐discharge. The morphology and (slight) structural differences are correlated with the synthesis chemistry itself (CO_3_^2−^ vs. OH^−^ coprecipitation) but also with the calcination conditions. Besides these different parameters (temperature, etc.), the lithiation agent LiOH melts before the lithiation of the precursor during calcination, as shown by thermal gravimetric analysis (TGA) and in situ XRD during calcination. Consequently, the liquid–solid reaction can induce different intermediates, which eventually can cause the respective stacking differences and finally increase Li^+^ intercalation resistances, thus decreasing the initial specific discharge capacities.

The decreased cycle life of LMR‐CO_3_ is finally concluded to relate to more detrimental Mn^3+/4^ redox due to more oxygen release (higher surface area and more voids). At the same time, impacts of the particle size distribution and/or surface differences can be possible, similar to different LiF content, shown by XPS and Warder titration, finally requiring a more detailed assessment for a systematic R&D.

## Funding

This study was supported by the LG Chem.

## Conflicts of Interest

The authors declare no conflicts of interest.

## Supporting information

Supplementary Material

## Data Availability

The data that support the findings of this study are available from the corresponding author upon reasonable request.

## References

[1] M. M. Thackeray , C. Wolverton , and E. D. Isaacs , “Electrical Energy Storage for Transportation—approaching the Limits of, and Going Beyond, Lithium‐Ion Batteries,” Energy & Environmental Science 5 (2012): 7854.

[2] S.‐T. Myung , F. Maglia , K.‐J. Park , et al., “Nickel‐Rich Layered Cathode Materials for Automotive Lithium‐Ion Batteries: Achievements and Perspectives,” ACS Energy Letters 2 (2017): 196–223.

[3] A. Butt , G. Ali , K. Tul Kubra , et al., “Recent Advances in Enhanced Performance of Ni‐Rich Cathode Materials for Li‐Ion Batteries: A Review,” Energy Technology 10 (2022): 2100775.

[4] J. U. Choi , N. Voronina , Y.‐K. Sun , and S.‐T. Myung , “Recent Progress and Perspective of Advanced High‐Energy Co‐Less Ni‐Rich Cathodes for Li‐Ion Batteries: Yesterday, Today, and Tomorrow,” Advanced Energy Materials 10 (2020): 2002027.

[5] J. Kasnatscheew , M. Evertz , R. Kloepsch , et al., “Learning From Electrochemical Data: Simple Evaluation and Classification of LiMO_2_ ‐Type‐Based Positive Electrodes for Li‐Ion Batteries,” Energy Technology 5 (2017): 1670–1679.

[6] H. Chen and C. Sun , “Recent Advances in Lithium‐Rich Manganese‐Based Cathodes for High Energy Density Lithium‐Ion Batteries,” Chemical Communications 59 (2023): 9029–9055. 37376977 10.1039/d3cc02195e

[7] M. Bettge , Y. Li , K. Gallagher , et al., “Voltage Fade of Layered Oxides: Its Measurement and Impact on Energy Density,” Journal of the Electrochemical Society 160 (2013): A2046–A2055.

[8] Y. Jin , Z. Zhao , P.‐G. Ren , et al., “Recent Advances in Oxygen Redox Activity of Lithium‐Rich Manganese‐Based Layered Oxides Cathode Materials: Mechanism, Challenges and Strategies,” Advanced Energy Materials 14, no. 40 (2024): 2402061.

[9] A. Arifiadi , S. Oster , D. Eum , et al., “Toward Practical Li‐Ion Cells With Li/Mn‐Rich Layered Oxide Cathodes: A Techno‐Economic Perspective on Material and Cell Design,” Advanced Science 13 (2026): e12467. 41028968 10.1002/advs.202512467 PMC12786379

[10] A. Arifiadi , T. Brake , F. Demelash , et al., “Toward High Specific Energy and Long Cycle Life Li/Mn‐Rich Layered Oxide || Graphite Lithium‐Ion Batteries via Optimization of Voltage Window,” Advanced Energy and Sustainability Research 5, no. 8 (2024):2400129.

[11] Z. Q. Deng and A. Manthiram , “Influence of Cationic Substitutions on the Oxygen Loss and Reversible Capacity of Lithium‐Rich Layered Oxide Cathodes,” The Journal of Physical Chemistry C 115 (2011): 7097–7103.

[12] D. Wang , I. Belharouak , G. M. Koenig , G. Zhou , and K. Amine , “Growth Mechanism of Ni_0.3_Mn_0.7_CO_3_ Precursor for High Capacity Li‐Ion Battery Cathodes,” Journal of Materials Chemistry 21 (2011): 9290.

[13] D. Wang , I. Belharouak , S. Gallagher , G. Zhou , and K. Amine , “Chemistry and Electrochemistry of Concentric ring Cathode Li_1.42_Ni_0.25_Mn_0.75_ O_2+γ_ for Lithium Batteries,” Journal of Materials Chemistry 22 (2012): 12039.

[14] S.‐H. Park , S.‐H. Kang , I. Belharouak , Y. K. Sun , and K. Amine , “Physical and Electrochemical Properties of Spherical Li_1+x_(Ni_1/3_Co_1/3_Mn_1/3_)_1−x_O_2_ Cathode Materials,” Journal of Power Sources 177 (2008): 177–183.

[15] R. De Palma , J. Kim , K. Driesen , J. Paulsen , and J. Hu , Carbonate Precursors for High Lithium and Maganese Containing Cathode Material, https://patents.google.com/patent/WO2015033246A2/es.

[16] R. De Palma , J. Kim , K. Driesen , J. Paulsen , and J. Hu , Carbonate Precursors for High Lithium and Manganese Containing Cathode Materials ‐ European Patent Office ‐ EP 3042409 B1, https://patents.google.com/patent/EP3042409B1/es.

[17] O. Cherkas , T. Beuvier , D. W. Breiby , Y. Chushkin , F. Zontone , and A. Gibaud , “Direct Observation of Microparticle Porosity Changes in Solid‐State Vaterite to Calcite Transformation by Coherent X‐Ray Diffraction Imaging,” Crystal Growth & Design 17 (2017): 4183–4188.

[18] S. H. R. Davies , and J. J. Morgan , “Manganese(II) Oxidation Kinetics on Metal Oxide Surfaces,” Journal of Colloid and Interface Science 129 (1989): 63–77.

[19] M.‐H. Lee , Y.‐J. Kang , S.‐T. Myung , and Y.‐K. Sun , “Synthetic Optimization of Li[Ni_1/3_Co_1/3_Mn_1/3_]O_2_ via Co‐Precipitation,” Electrochimica Acta 50 (2004): 939–948.

[20] M. Schrimpf , J. A. Esteban , H. Warmeling , T. Färber , A. Behr , and A. J. Vorholt , “Taylor‐Couette Reactor: Principles, Design, and Applications,” AIChE Journal 67, no. 5 (2021): e17228.

[21] J. Pattanayak , V. S. Rao , and H. S. Maiti , “Preparation and Thermal Stability of Manganese Oxides Obtained by Precipitation From Aqueous Manganese Sulphate Solution,” Thermochimica Acta 153 (1989): 193–204.

[22] O. J. Borkiewicz , B. Shyam , K. M. Wiaderek , C. Kurtz , P. J. Chupas , and K. W. Chapman , “The AMPIX Electrochemical Cell: A Versatile Apparatus for In Situ X‐Ray Scattering and Spectroscopic Measurements,” Journal of Applied Crystallography 45 (2012): 1261–1269.

[23] J. Medema and J. P. W. Houtman , “Brunauer‐Emmett‐Teller Specific Measurement of Solids Using Krypton,” Analytical and Bioanalytical Chemistry 41, no. 1 (1969): 209‐211.

[24] M. Thommes , K. Kaneko , A. V. Neimark , et al., “Physisorption of Gases, With Special Reference to the Evaluation of Surface Area and Pore Size Distribution (IUPAC Technical Report),” Pure and Applied Chemistry 87 (2015):1051–1069.

[25] N. Fairley , V. Fernandez , M. Richard‐Plouet , et al., “Systematic and Collaborative Approach to Problem Solving Using X‐Ray Photoelectron Spectroscopy,” Applied Surface Science Advances 5 (2021): 100112.

[26] A. R. Schuer , M. Kuenzel , S. Yang , et al., “Diagnosis Tools for Humidity‐Born Surface Contaminants on Li[Ni_0.8_Mn_0.1_Co_0.1_]O_2_ Cathode Materials for Lithium Batteries,” Journal of Power Sources 525 (2022): 231111.

[27] D. Massiot , F. Fayon , M. Capron , et al., “Modelling One‐ and Two‐Dimensional Solid‐State NMR Spectra,” Magnetic Resonance in Chemistry 40 (2002): 70–76.

[28] E. Trevisanello , R. Ruess , G. Conforto , F. H. Richter , and J. Janek , “Polycrystalline and Single Crystalline NCM Cathode Materials—Quantifying Particle Cracking, Active Surface Area, and Lithium Diffusion,” Advanced Energy Materials 11 (2021): 2003400.

[29] M. J. Lüther , S.‐K. Jiang , M. A. Lange , et al., “Systematic “Apple‐to‐Apple” Comparison of Single‐Crystal and Polycrystalline Ni‐Rich Cathode Active Materials: From Comparable Synthesis to Comparable Electrochemical Conditions,” Small Structures 5, no. 11 (2024): 2400119.

[30] S. A. Hassanzadeh‐Tabrizi , “Precise Calculation of Crystallite Size of Nanomaterials: A Review,” Journal of Alloys and Compounds 968 (2023): 171914.

[31] H. Liu , W. Hua , S. Kunz , et al., “Tailoring Superstructure Units for Improved Oxygen Redox Activity in Li‐Rich Layered Oxide Battery’s Positive Electrodes,” Nature Communications 15 (2024): 9981. 10.1038/s41467-024-54312-z PMC11573992 39557874

[32] J. Bréger , M. Jiang , N. Dupré , et al., “High‐Resolution X‐Ray Diffraction, DIFFaX, NMR and First Principles Study of Disorder in the Li_2_MnO_3_–Li[Ni_1/2_Mn_1/2_]O_2_ Solid Solution,” Journal of Solid State Chemistry 178 (2005): 2575–2585.

[33] C. P. Grey and Y. J. Lee , “Lithium MAS NMR Studies of Cathode Materials for Lithium‐Ion Batteries,” Solid State Sciences 5 (2003): 883–894.

[34] H. Yang , L. Wang , Y. Li , et al., “Co‐Free Gradient Lithium‐Rich Cathode for High‐Energy Batteries With Optimized Cyclability,” Proceedings of the National Academy of Sciences of the United States of America 121 (2024):e2412460121. 39630874 10.1073/pnas.2412460121 PMC11648880

[35] F. Sigel , B. Schwarz , K. Kleiner , et al., “Thermally Induced Structural Reordering in Li‐ and Mn‐Rich Layered Oxide Li Ion Cathode Materials,” Chemistry of Materials 32 (2020): 1210–1223.

[36] X. Li , M. Tang , X. Feng , et al., “Lithiation and Delithiation Dynamics of Different Li Sites in Li‐Rich Battery Cathodes Studied by Operando Nuclear Magnetic Resonance,” Chemistry of Materials 29 (2017): 8282–8291.

[37] L. Stolz , M. Gaberšček , M. Winter , and J. Kasnatscheew , “Different Efforts but Similar Insights in Battery R&D: Electrochemical Impedance Spectroscopy *vs* Galvanostatic (Constant Current) Technique,” Chemistry of Materials 34 (2022): 10272–10278.

[38] J. Kasnatscheew , U. Rodehorst , B. Streipert , et al., “Learning From Overpotentials in Lithium Ion Batteries: A Case Study on the LiNi_1/3_Co_1/3_Mn_1/3_ O_2_ (NCM) Cathode,” Journal of the Electrochemical Society 163 (2016): A2943–A2950.

[39] M. Merz , B. Ying , P. Nagel , S. Schuppler , and K. Kleiner , “Reversible and Irreversible Redox Processes in Li‐Rich Layered Oxides,” Chemistry of Materials 33 (2021): 9534–9545.

[40] X. Liu , Q. Zhang , J. Ji , and F. Lian , “Jointly Improving Anionic‐Cationic Redox Reversibility of Lithium‐Rich Manganese‐Based Cathode Materials by N Surface Doping,” ACS Applied Materials & Interfaces 16 (2024): 42966–42975. 39023357 10.1021/acsami.4c08840

[41] W. Mei , H. Chen , J. Sun , and Q. Wang , “The Effect of Electrode Design Parameters on Battery Performance and Optimization of Electrode Thickness Based on the Electrochemical–thermal Coupling Model,” Sustainable Energy & Fuels 3 (2019): 148–165.

[42] J. Smekens , R. Gopalakrishnan , N. Van den Steen , et al., “Influence of Electrode Density on the Performance of Li‐Ion Batteries: Experimental and Simulation Results,” Energies 9 (2016):104.

[43] L. Lan , Q. Li , G. Gu , H. Zhang , and B. Liu , “Hydrothermal Synthesis of γ‐MnOOH Nanorods and Their Conversion to MnO_2_, Mn_2_O_3_, and Mn_3_O_4_ Nanorods,” Journal of Alloys and Compounds 644 (2015): 430–437.

[44] G. M. Busse , P. M. Csernica , K. Lim , et al., “Calcination Heterogeneity in Li‐Rich Layered Oxides: A Systematic Study of Li_2_CO_3_ Particle Size,” Chemistry of Materials 35 (2023): 10658–10671.

[45] Y. Chen , S. Luo , J. Leng , et al., “Exploring the Synthesis Conditions and Formation Mechanisms of Li‐Rich Layered Oxides via Solid‐State Method,” Journal of Alloys and Compounds 854 (2021): 157204.

[46] W. Hua , K. Wang , M. Knapp , et al., “Chemical and Structural Evolution During the Synthesis of Layered Li(Ni, Co, Mn)O_2_ Oxides,” Chemistry of Materials 32 (2020): 4984–4997.

[47] C. Xu , A. J. Merryweather , S. S. Pandurangi , et al., “Operando Visualization of Kinetically Induced Lithium Heterogeneities in Single‐Particle Layered Ni‐Rich Cathodes,” Joule 6 (2022): 2535–2546.

[48] D. Mohanty , S. Kalnaus , R. A. Meisner , et al., “Structural Transformation of a Lithium‐Rich Li_1.2_Co_0.1_Mn_0.55_Ni_0.15_O_2_ Cathode During High Voltage Cycling Resolved by In Situ X‐Ray Diffraction,” Journal of Power Sources 229 (2013): 239–248.

[49] J. Li , W. Li , C. Zhang , et al., “Tuning Li_2_MnO_3_‐Like Domain Size and Surface Structure Enables Highly Stabilized Li‐Rich Layered Oxide Cathodes,” ACS Nano 17 (2023): 16827–16839. 37582222 10.1021/acsnano.3c03666

[50] W. Li , H. Y. Asl , Q. Xie , and A. Manthiram , “Collapse of LiNi_1‐ x‐ y_ Co_x_Mn_y_O_2_ Lattice at Deep Charge Irrespective of Nickel Content in Lithium‐Ion Batteries,” Journal of the American Chemical Society 141 (2019): 5097–5101. 30887807 10.1021/jacs.8b13798

[51] M. Li , X. Yang , X. Wei , Y. Zhu , X. Sun , and M. D. Gu , “Cathode‐Electrolyte Interphase of Ni‐Rich Layered Oxides: Evolving Structure and Implication on Stability,” Nano Letters 25 (2025):2769–2776. 39932008 10.1021/acs.nanolett.4c05838

[52] J. Kasnatscheew , M. Evertz , B. Streipert , et al., “The Truth About the 1st Cycle Coulombic Efficiency of LiNi_1/3_Co_1/3_Mn_1/3_O_2_ (NCM) Cathodes,” Physical Chemistry Chemical Physics 18 (2016): 3956–3965. 26771035 10.1039/c5cp07718d

[53] J. Kasnatscheew , B. Streipert , S. Röser , R. Wagner , I. Cekic Laskovic , and M. Winter , “Determining Oxidative Stability of Battery Electrolytes: Validity of Common Electrochemical Stability Window (ESW) Data and Alternative Strategies,” Physical Chemistry Chemical Physics 19 (2017): 16078–16086. 28597888 10.1039/c7cp03072j

[54] B. Streipert , L. Stolz , G. Homann , et al., “Conventional Electrolyte and Inactive Electrode Materials in Lithium‐Ion Batteries: Determining Cumulative Impact of Oxidative Decomposition at High Voltage,” ChemSusChem 13 (2020):5301–5307. 32692891 10.1002/cssc.202001530 PMC7589409

[55] J. Kasnatscheew , R. Wagner , M. Winter , and I. Cekic‐Laskovic , “Interfaces and Materials in Lithium Ion Batteries: Challenges for Theoretical Electrochemistry,” Topics in Current Chemistry, 376, no. 16 (2018):23‐51. 29671099 10.1007/s41061-018-0196-1

[56] S. Klein , P. Barmann , O. Fromm , et al., “Prospects and Limitations of Single‐Crystal Cathode Materials to Overcome Cross‐Talk Phenomena in High‐Voltage Lithium Ion Cells,” Journal of Materials Chemistry A 9 (2021):7546–7555.

