# Supplementary material for: Carbonate‐ Versus Hydroxide‐Based Precursor for the Synthesis of Li/Mn Rich Cathodes: Exploring Differences in Discharge Capacities and Cycle Life
Source: Small Sci. 2026 Aug 12;6(8):e70370. doi: 10.1002/smsc.70370 (PMC13489277; doi:10.1002/smsc.70370)
Supplement: Supplementary file 1 [file SMSC-6-e70370-s001.pdf]

Supporting Information:

Carbonate- *vs.* Hydroxide-based Precursor for the Synthesis of Li/Mn Rich Cathodes: Exploring Differences in Discharge Capacities and Cycle Life

*Pranti Sutar [a], Johannes Helmut Thienenkamp [c], Laurin Profanter [a], Tim Messink [a], Annalena Krude [c], Uta Rodehorst [a], Hyuck Hur [b], Gunther Brunklaus [c], Dominik Voigt [a], Martin Winter [a,c], Johannes Kasnatscheew [a], **

[a:] MEET Battery Research Center, Institute of Physical Chemistry, University of Münster, Corrensstraße 46, 48149 Münster, Germany

[b:] LG Energy Solution, 128 Yeoui-daero, Yenondeungpo District, Seoul, South Korea

[c:] Helmholtz-Institute Münster, IMD-4, Forschungszentrum Jülich GmbH, Corrensstraße 46, 48149 Münster, Germany

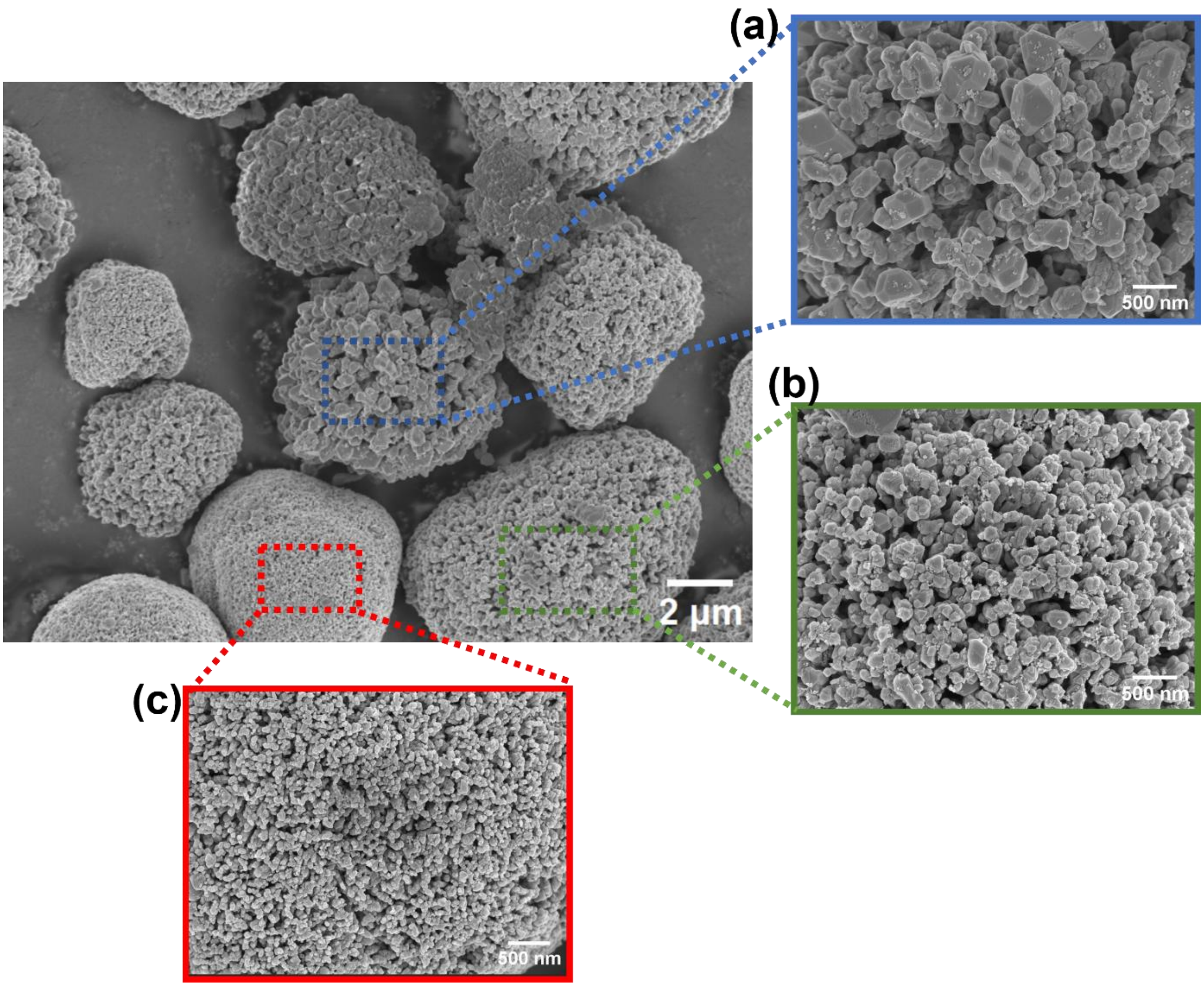

Figure.S1: SEM images of LMR-CO_3 active material showcasing varing primary particle size distribution

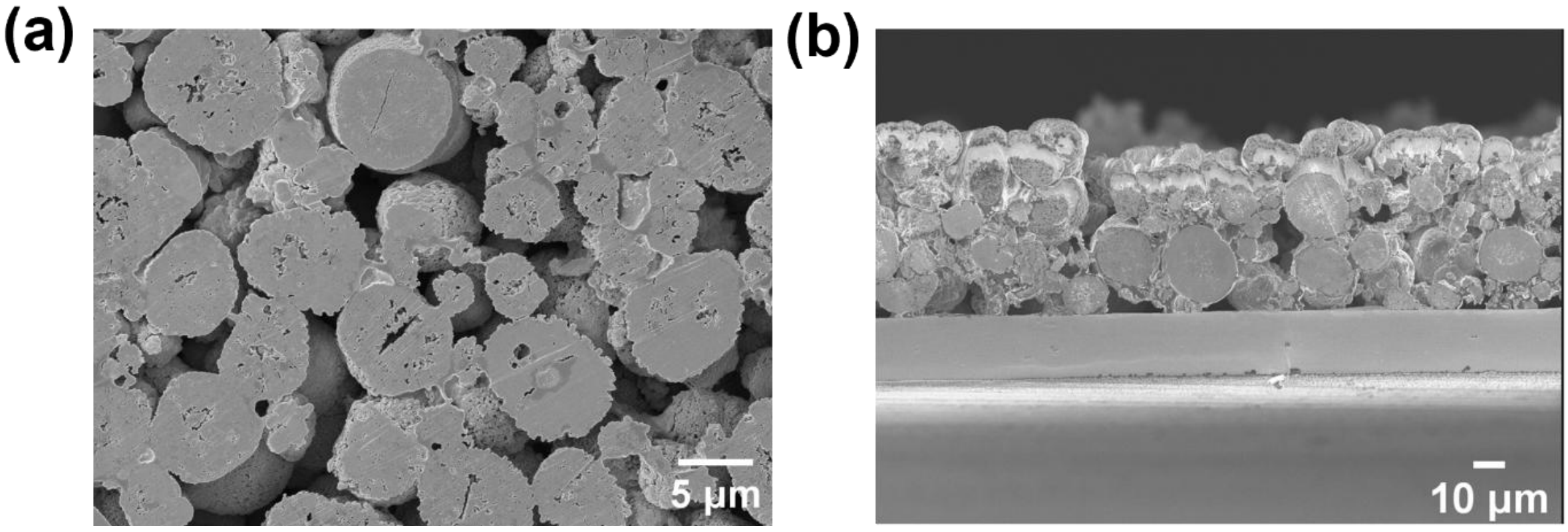

Figure. S2: Cross-section SEM images of (a)LMR-CO_3 and (b) LMR-$(OH)_2$.

Table S1: ICP-OES stoichiometry data.

**Samples**	**Ni: Mn**	**Li: *TM***
LMR-CO_3	35:65	1.15
LMR-$(OH)_2$	35:65	1.13

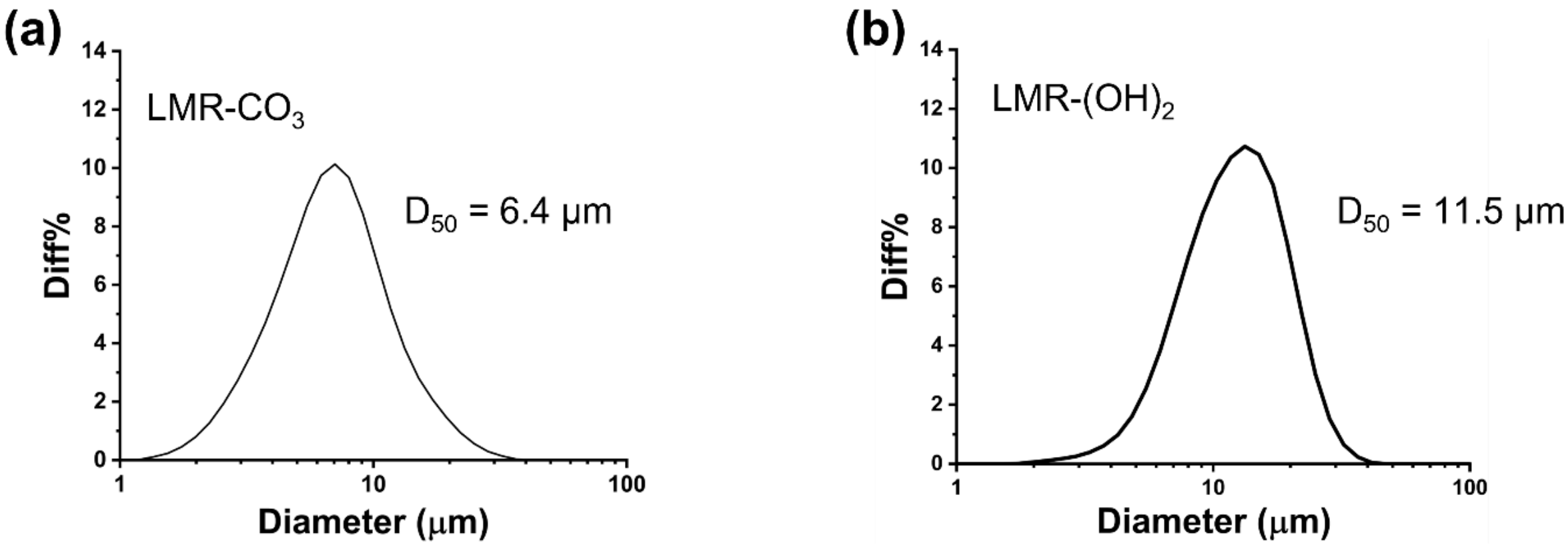

Figure S3: Particle size distribution.

Table S2: Rietveld refinement parameters and results assuming $R\bar{3}m$ space group

For LMR-CO3

Sample	*a,b* (Å)	c (Å)	Volume ($Å^3$)	Cell density (g/cm^3)	R_{bragg}	R_{exp}	R_{wp}	R_p	GOF
**$LMR-CO_3$**	**2.869(9)**	**14.268(1)**	**101.74(1)**	**4.367**	**1.66**	**2.01**	**5.39**	**3.71**	**2.67**

Atom	Wayckoff position	x	y	z	Occupancy
Li	3b	0	0	0.5	0.941(2)
Ni	3a	0	0	0	0.221(5)
Mn	3a	0	0	0	0.6
Li	3a	0	0	0	0.07
O	6c	0	0	256(9)	1
Ni (mixing)	3b	0	0	0.5	0.058(2)
Li (mixing)	3a	0	0	0	0.098 (5)

For LMR-(OH)2:

Sample	*a,b* (Å)	c (Å)	Volume ($Å^3$)	Cell density (g/cm^3)	R_{bragg}	R_{exp}	R_{wp}	R_p	GOF
**$LMR-(OH)_2$**	**2.868(1)**	**14.270(1)**	**101.66(1)**	**4.33(1)**	**1.54**	**1.98**	**5.02**	**3.57**	**2.53**

Atom	Wayckoff position	x	y	z	Occupancy
Li	3b	0	0	0.5	0.953(7)
Ni	3a	0	0	0	0.275(6)
Mn	3a	0	0	0	0.6
Li	3a	0	0	0	0.07
O	6c	0	0	256(4)	1
Ni (mixing)	3b	0	0	0.5	0.046(3)
Li (mixing)	3a	0	0	0	0.014(3)

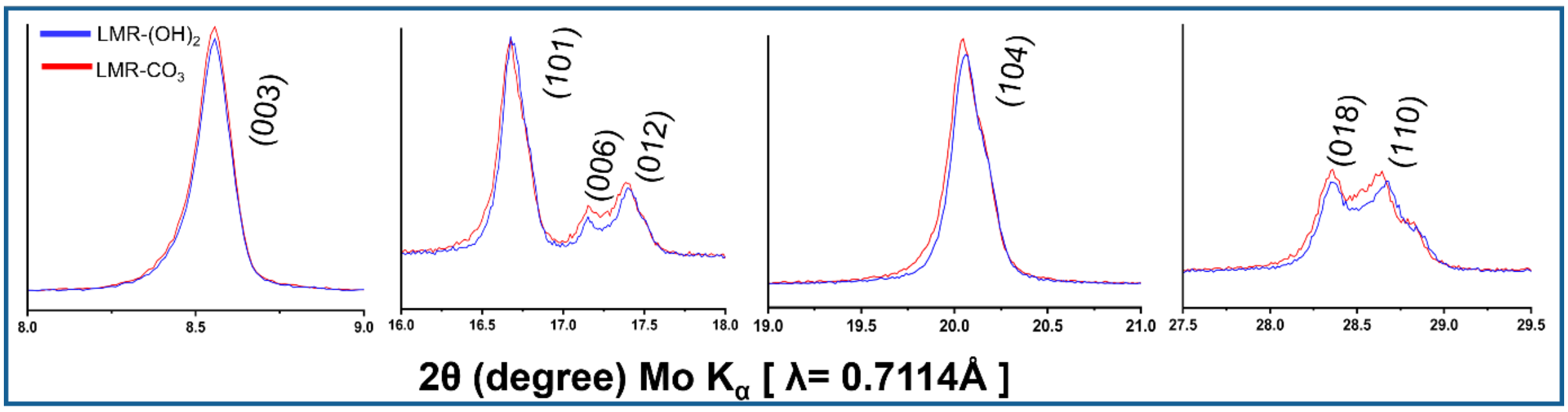

Figure S4: Reflection shift and line broadening.

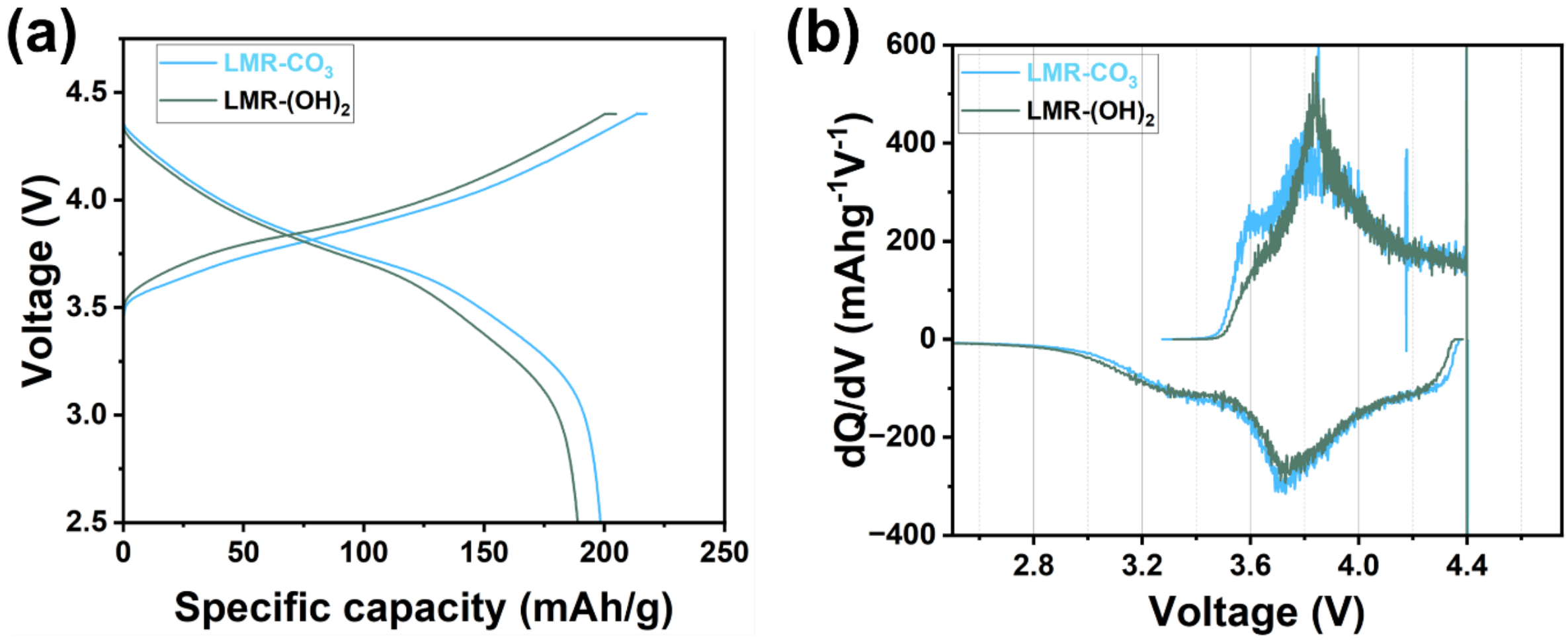

Figure S5: Comparison 2nd cycle after formation cycle run at 20 °C within the voltage range of 4.4 V – 2.5 V against Li metal at 0.1C . (a) voltage profile of both LMR showcasing the differences in capacity upon further cycling after formation cycle, (b) dQ/dV vs V plot of 2nd cycle.

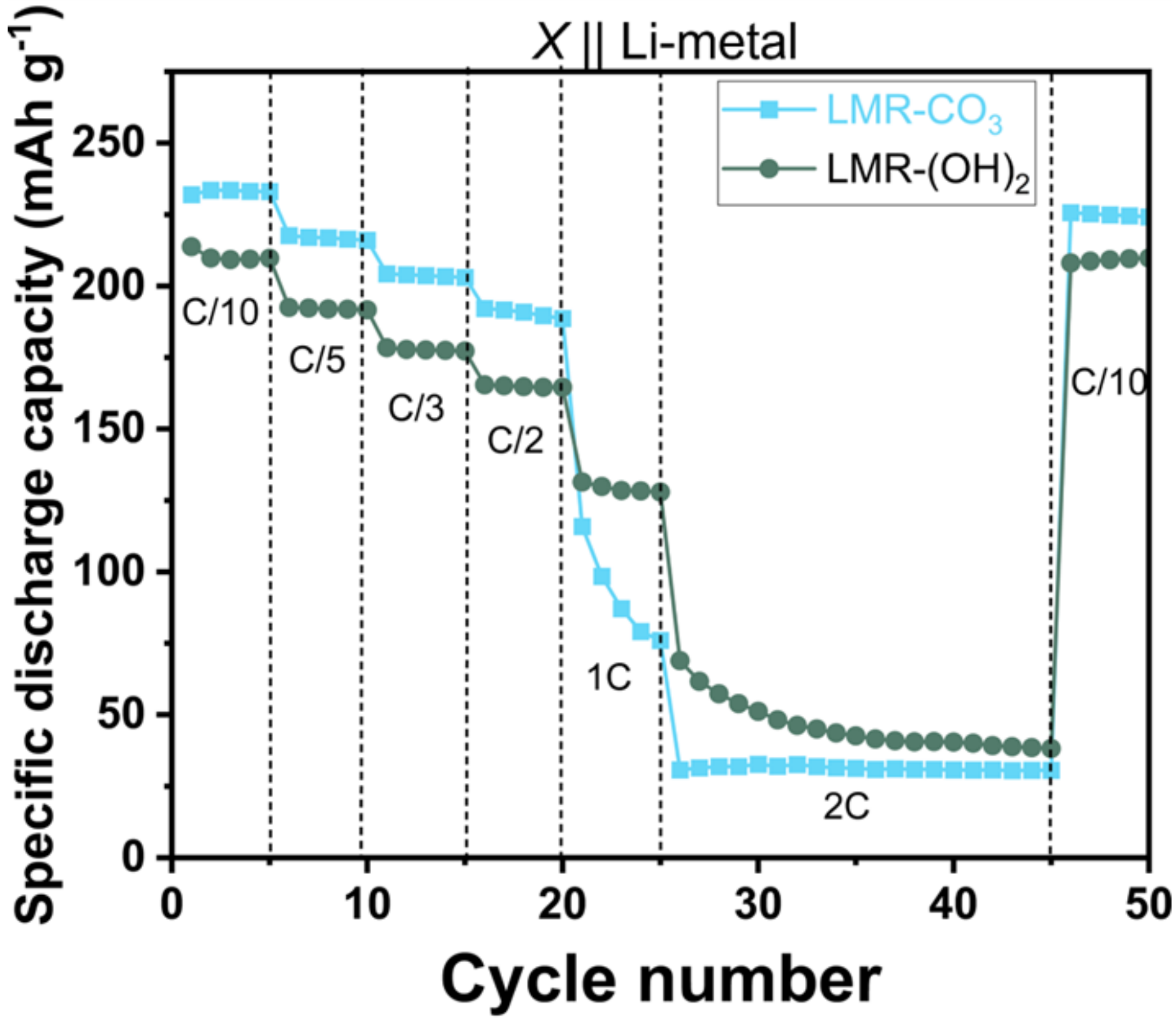

Figure S6: Rate performance analysis at different C-rate at 20 °C

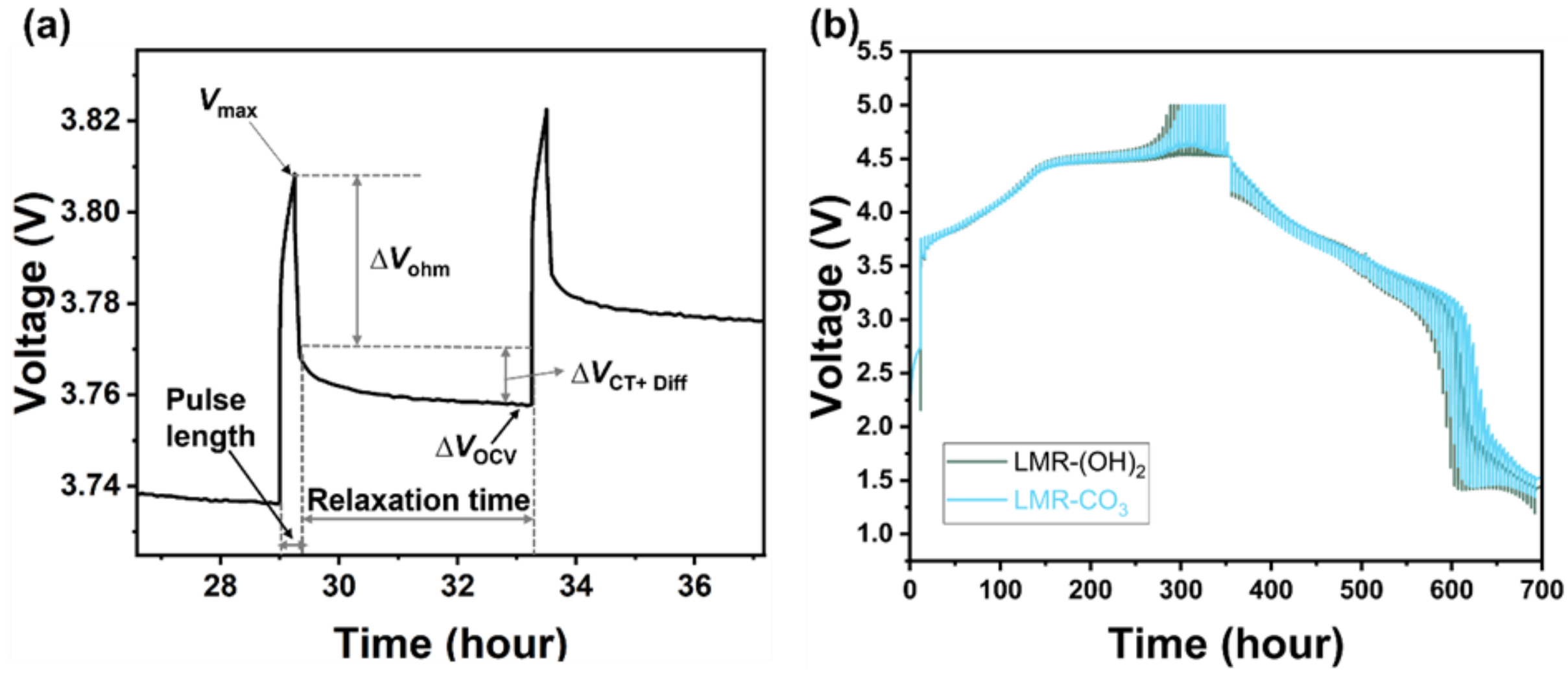

Figure S7: (a) principle and (b) data from Galvanostatic intermittent titration technique (GITT).

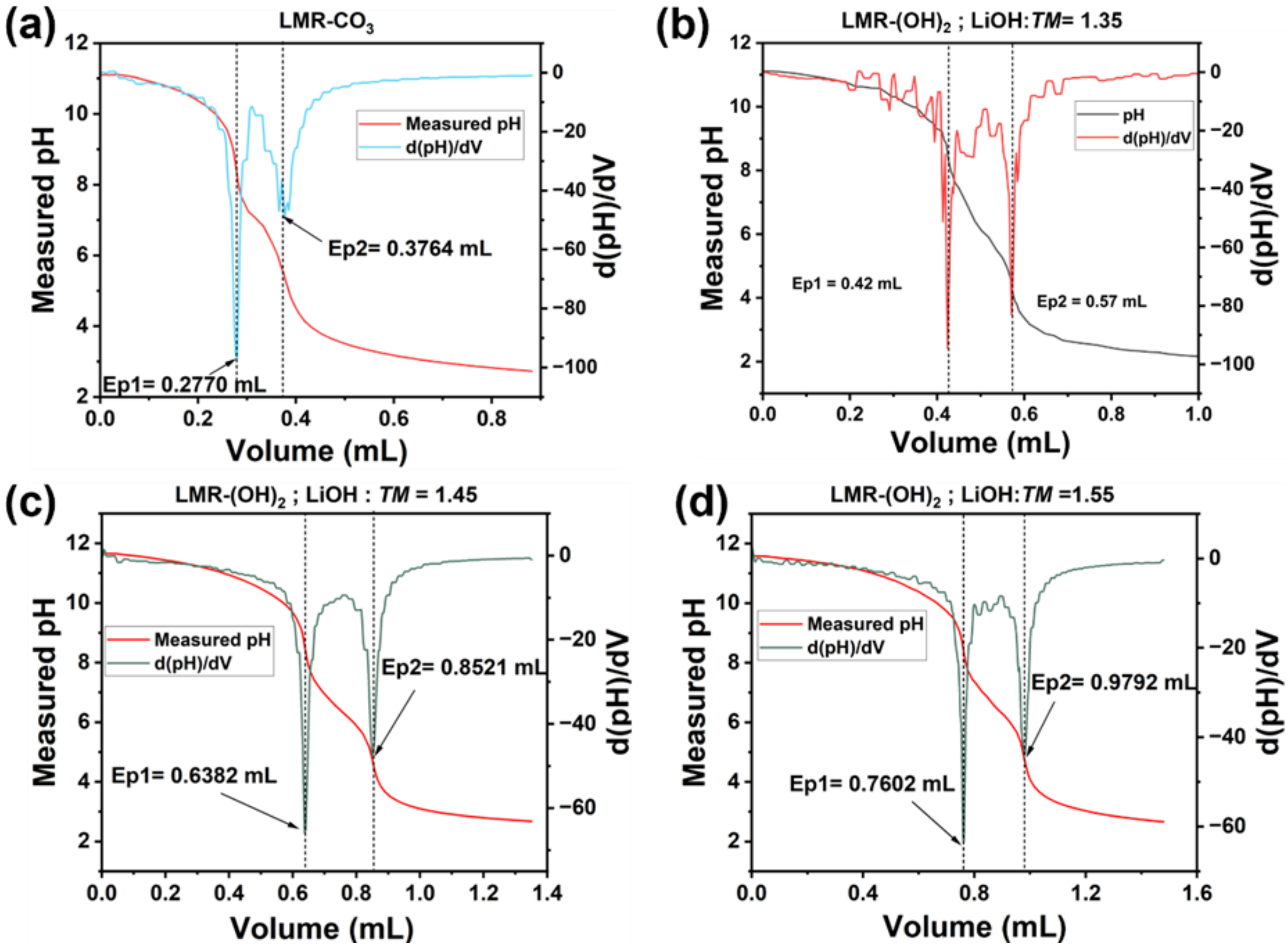

Figure S8: Warder titration.

Table S3: Warder titration curve (pH vs volume) along with their differential curve (d(pH) vs volume) of (a)LMR-CO3, and LMR-(OH)2 having LiOH:TM ratio of (b) 1.35, (c) 1.45 and (d) 1.55. In table 3 weight percentage wt(%) of LiOH and Li2CO3 of all the materials given.

Sample	Wt% of LiOH	Wt% Li_2CO_3
LMR-CO_3	0.1301	0.1668
LMR-$(OH)_2$ (Li/TM= 1.35)	0.2265	0.2217
LMR-$(OH)_2$ (Li/TM= 1.45)	0.3483	0.3171
LMR-$(OH)_2$ (Li/TM= 1.55)	0.4517	0.3288

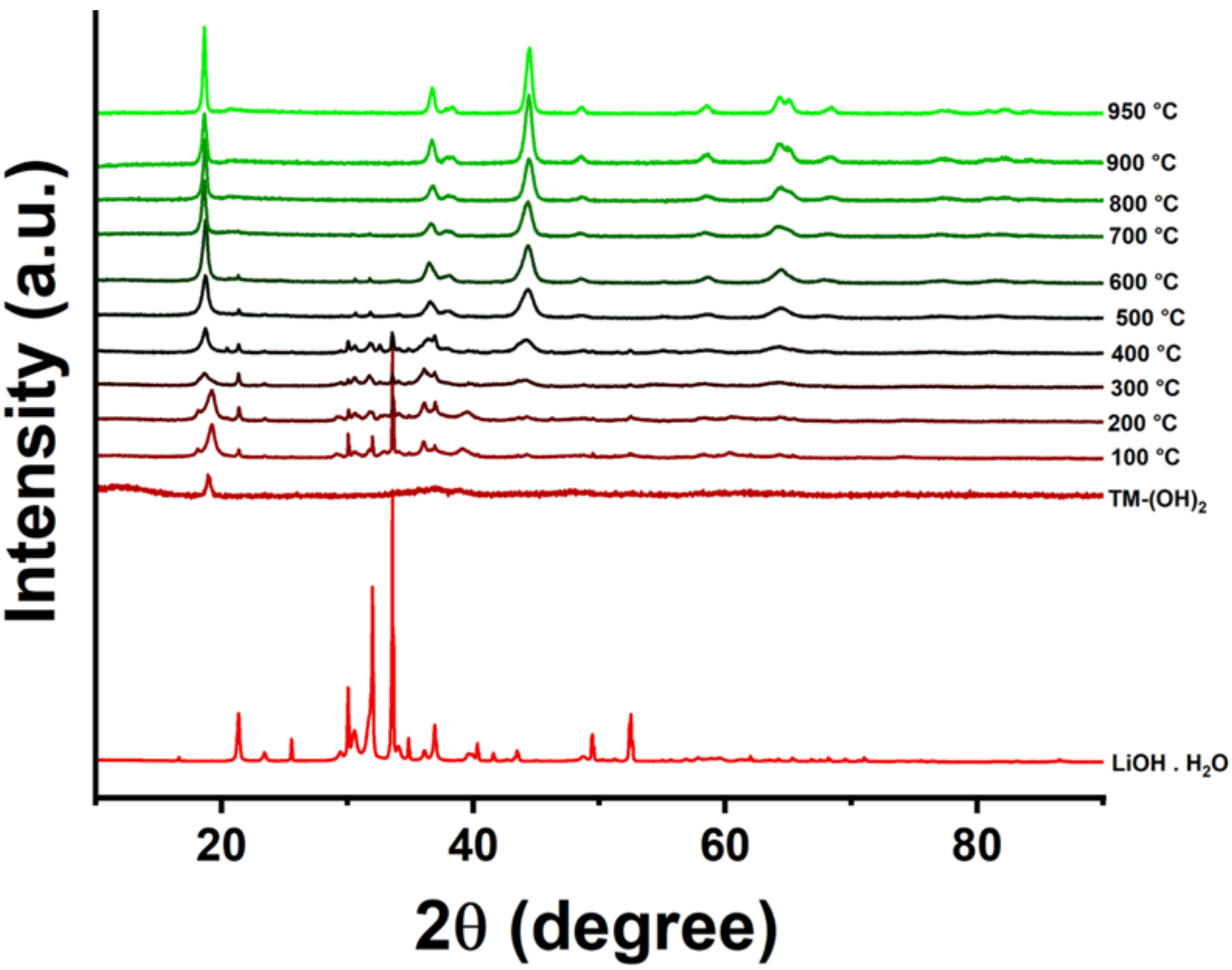

Figure S9: Ex-situ calcination XRD of mixture of TM-$(OH)_2$ and LiOH · H_2O showcasing the structural evolution of LMR-(OH)2. The spinel phase is absent here which is visible in in-situ experiment.

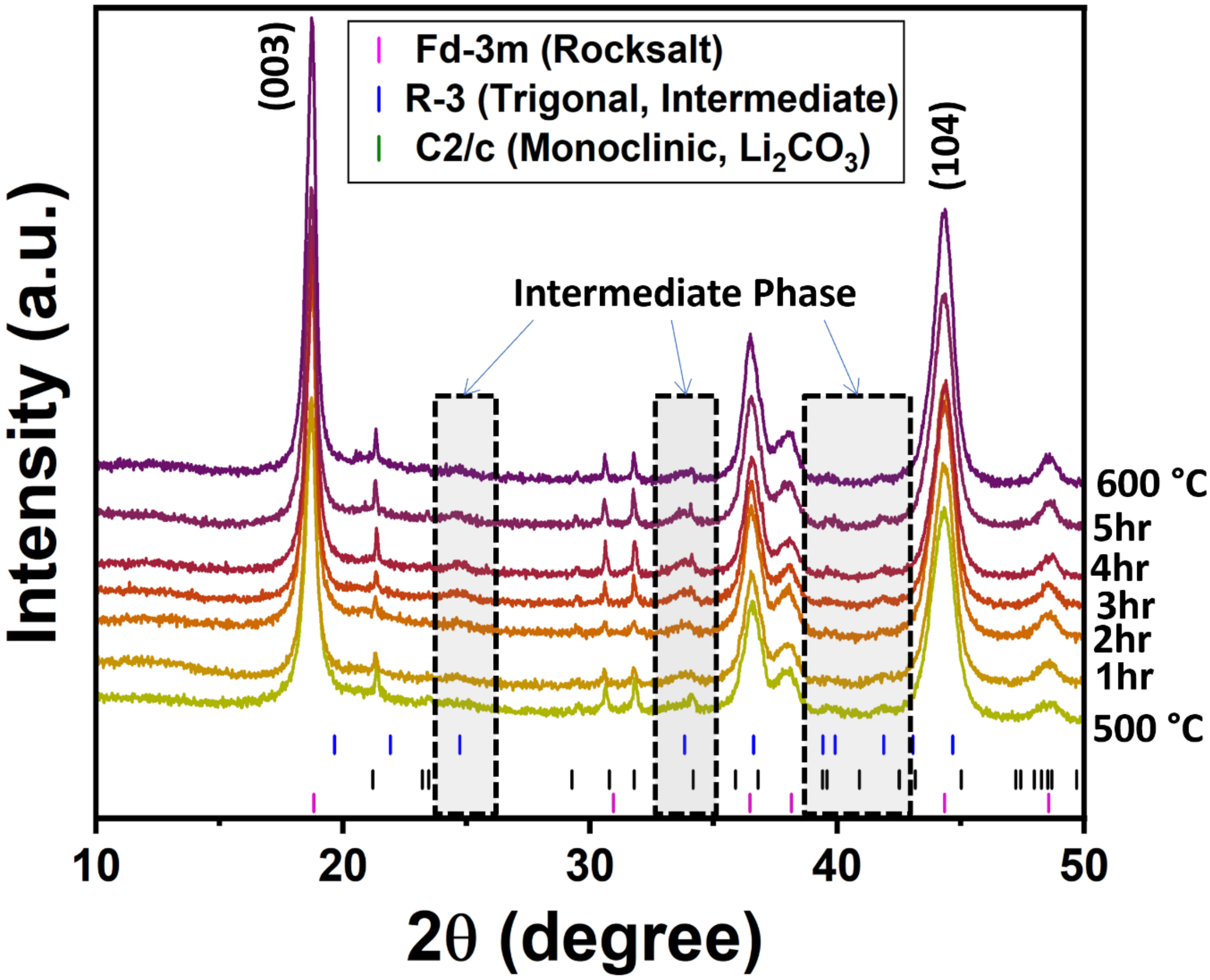

Figure S10: Ex-situ XRD pattern of calcination at 500 °C for a varied duration and 600 °C without duration. Before measurement samples are directly quenched with liquid N2. We have index reflections of an intermediate phase visible after 1hr hold at 500 °C, indicating this is a pseudo layered phase.

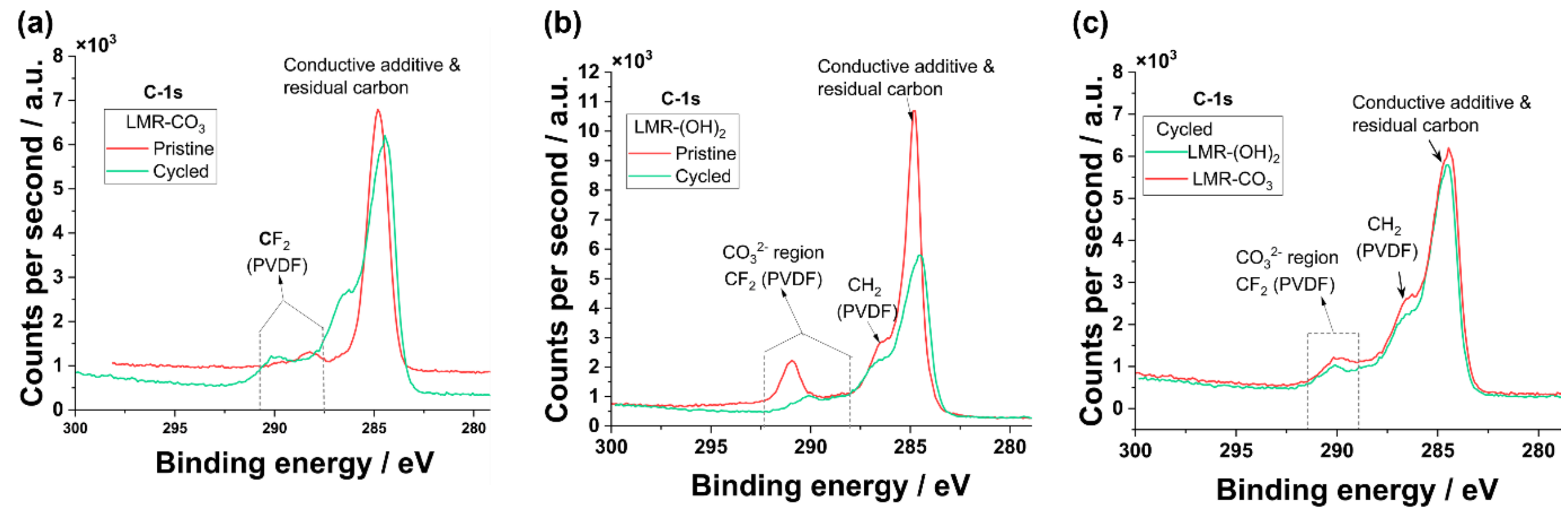

Figure S11: XPS spectra of C-1s of pristine and cycled electrodes of (a)LMR-CO_3., (b) LMR-$(OH)_2$ and (c) overlay of cycled electrodes.

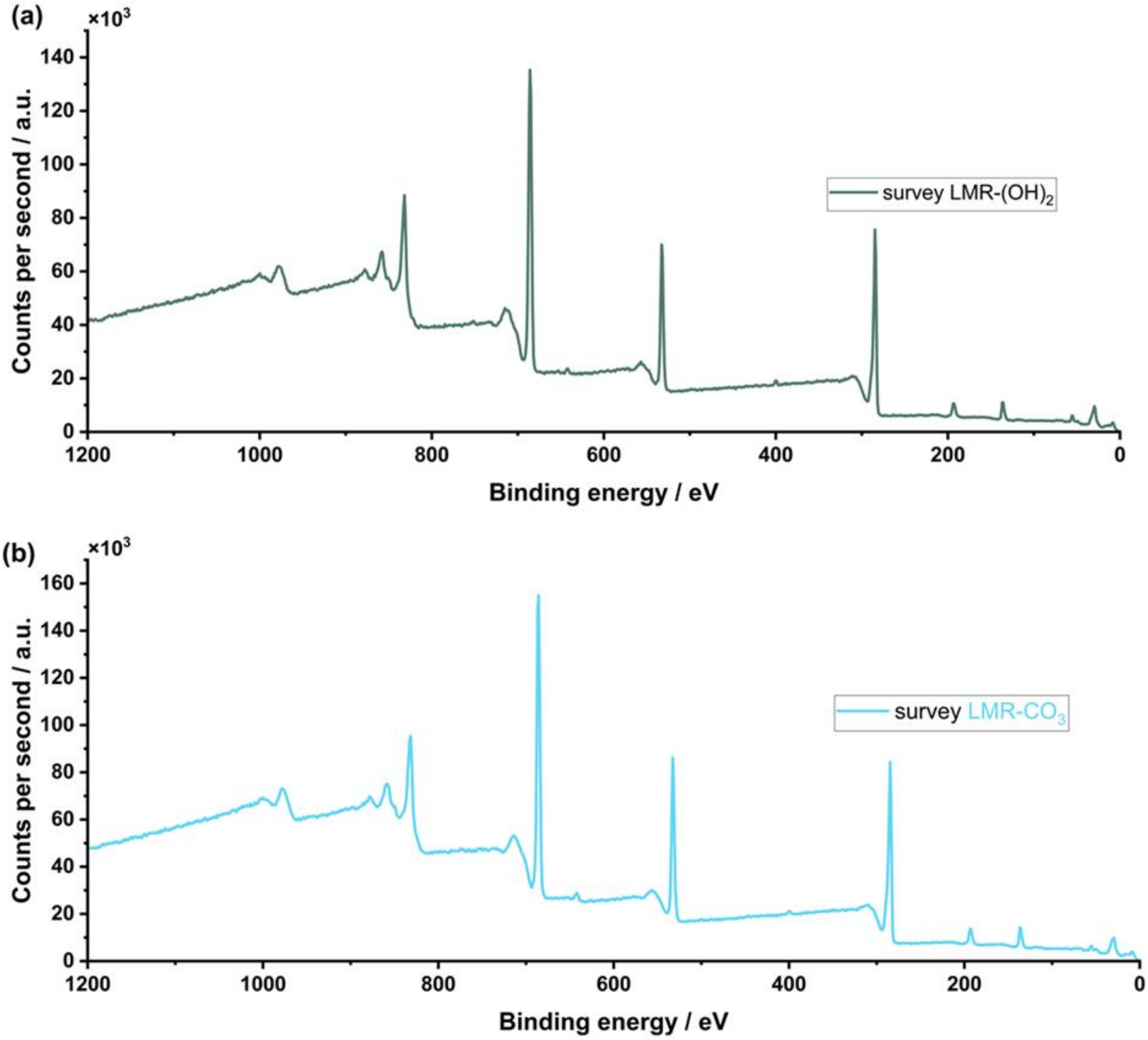

Figure S12: XPS survey scan.

S13: ^{7}Li-MAS NMR of LMR Cathodes:

To deconvolute the ^{7}Li MAS NMR spectra of LMR-CO_3, at least three isotropic resonances are required (denoted as green, orange, blue). The corresponding spinning sidebands are separated from the isotropic peaks by the MAS rate and were only changed in amplitude upon spectral deconvolution. All the signals are represented by patterns, where the isotropic peaks were optimized with respect to their amplitudes, line widths, chemical shifts and Gauss/Lorentz ratios, thereby resulting in a total number of 20 fitting parameters. The fitted data and the actual spectra are shown in red and black, respectively. A similar deconvolution protocol was used for ^{7}Li NMR spectra of LMR-$(OH)_2$, but adding an isotropic peak at 0ppm, reflecting the presence of residual precursor(s), and increasing the total number of fitting parameters to 22. While the relative intensity of the residual precursor is about 1.1%, the relative intensity of the resonance at 548ppm amounts to 55.6%. The summed intensity of resonances at chemical shifts >700ppm is 43.3%, resulting in an intensity ratio of 1.28:1. In comparison, the intensity ratio of Li-ion sites with more elongated Li-O-Mn (<700ppm) and more perpendicular bond angles (>700ppm) is 0.62:1.

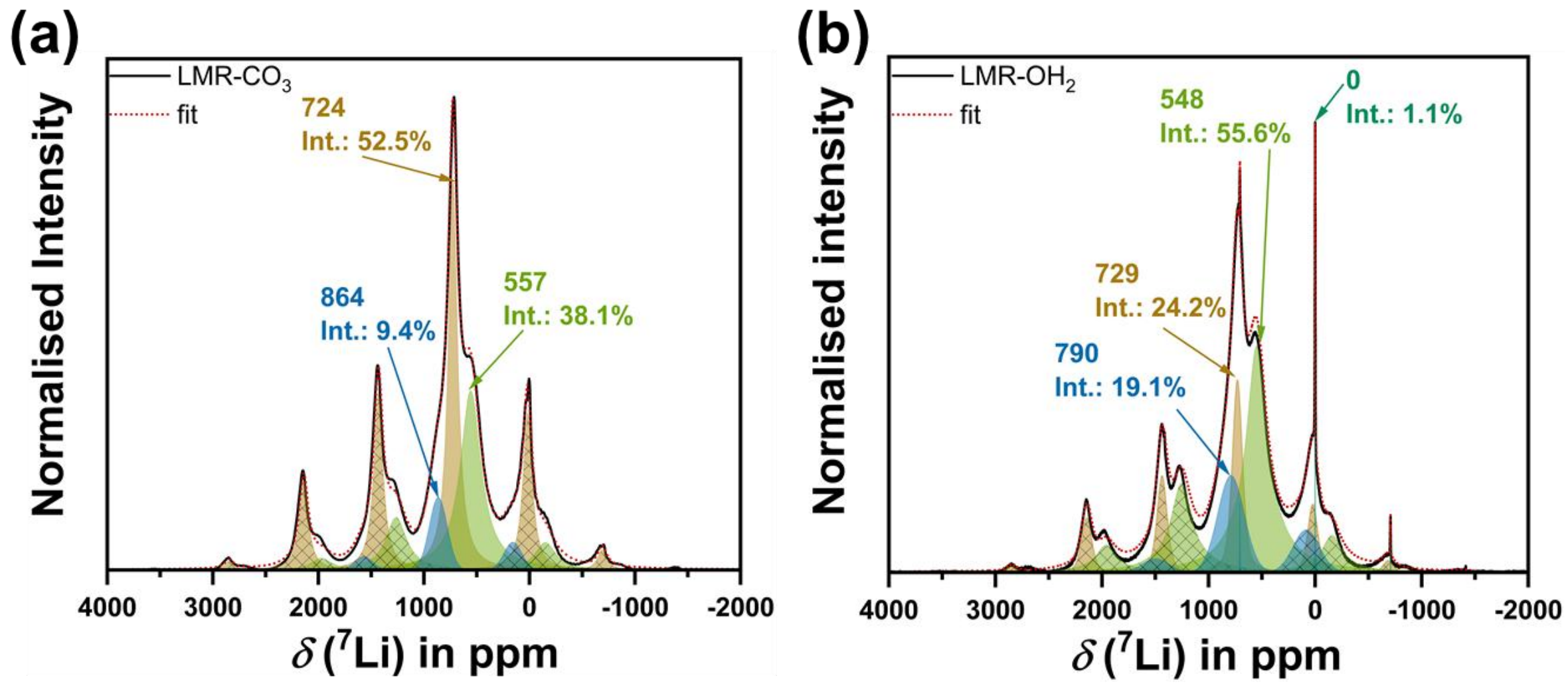

Figure S13: Deconvoluted ^{7}Li MAS NMR spectra of (a) LMR-CO3 and (b) LMR-(OH)2, respectively. Three isotropic resonances at 557ppm (green), 724ppm (orange) and 864ppm (blue) are used to fit the ^{7}Li spectrum of LMR-CO3, while four isotropic resonances at 0ppm (turquoise), 548ppm (green), 729ppm (orange) and 790ppm (blue) are required for the ^{7}Li spectrum of LMR-(OH)2. The rotational sidebands of the isotropic peaks have the same colours as the isotropic peaks, but are filled with pattern. the overall fits are coloured in red.

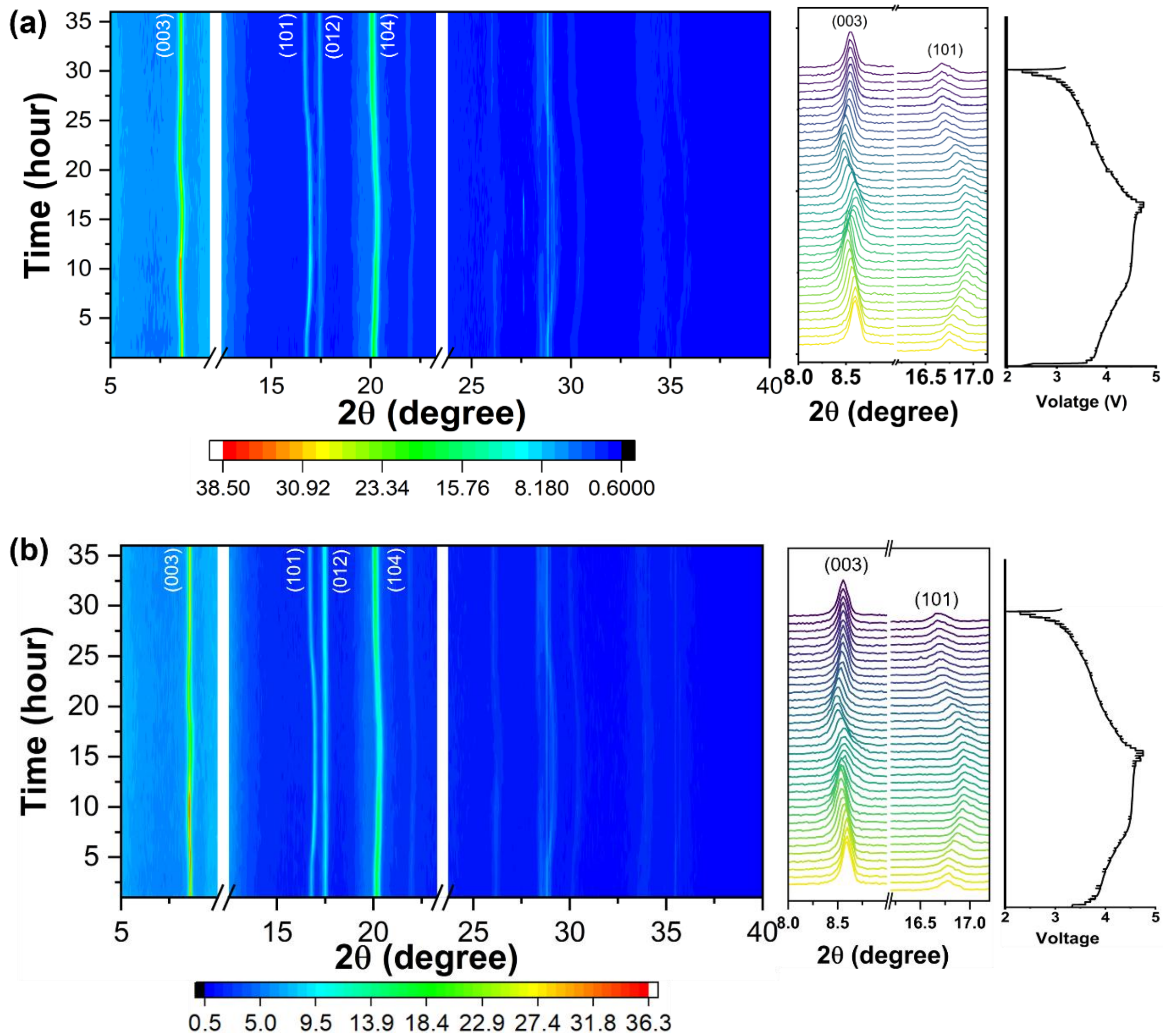

Figure S14: Operando XRD pattern of (a) LMR-CO_3 and (b) LMR-$(OH)_2$

S15 In-house build modified AMPIX cell:

With the inspiration from Argonne's multipurpose *in-situ* X-ray (AMPIX) cell design, we have designed a cell compatible with our XRD set up in transmission mode. The cell body is made up of polyetheretherketone (PEEK) which is known for its strength, stiffness, chemically and electronically insulating properties. This material perfect for cell body because it helps from any short circuit when in contact with other metal. The bottom and the top electrodes are made up of stainless steel, also, fitted with rigid X-ray transmission window made of glassy carbon. A rubber gasket goes in between the top and bottom electrodes, within the gasket the battery stack sits. A cross-section image of close cell is shown in figure S17b and close cell in real image shown in figure S17c..

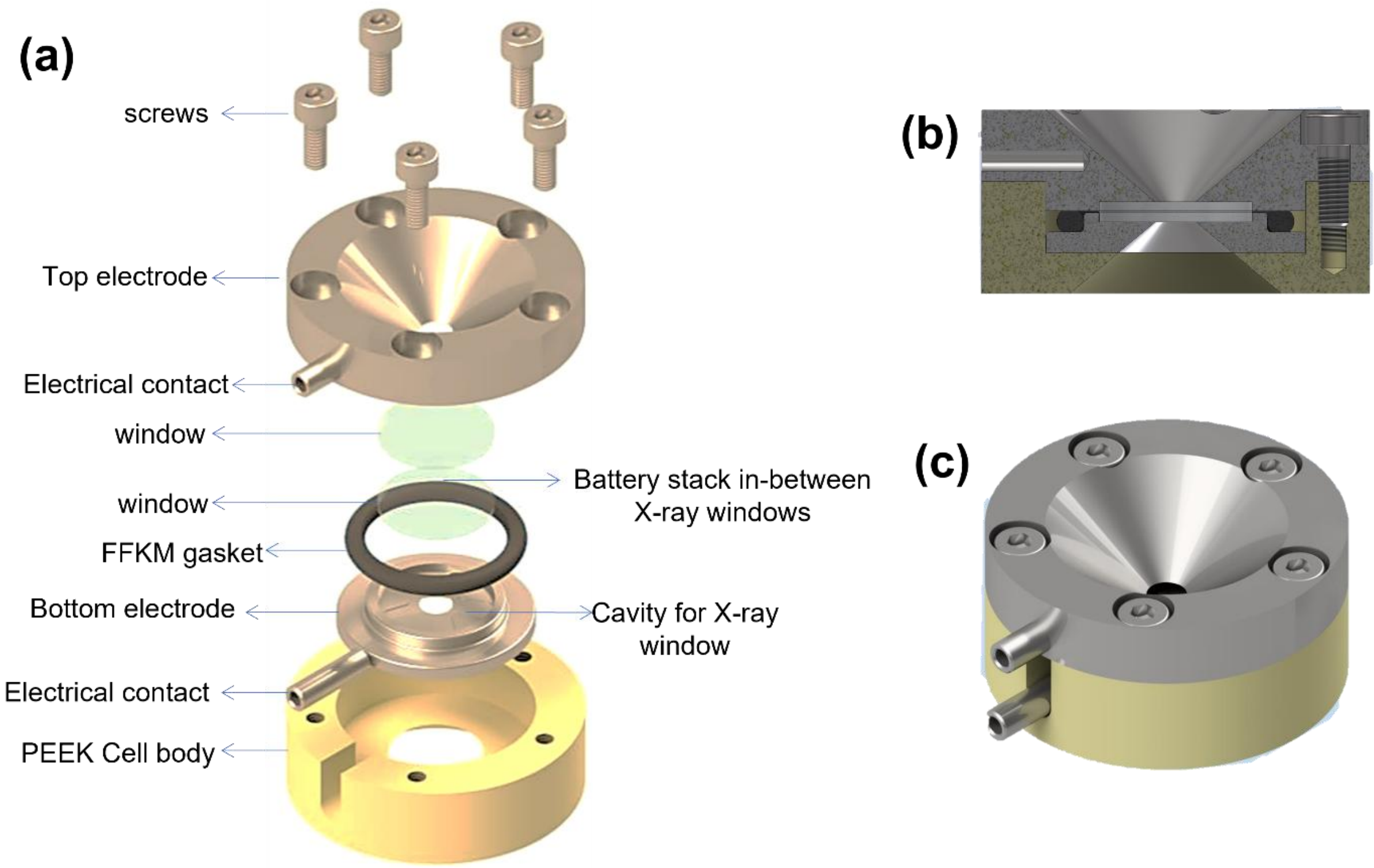

Figure S15: (a) expanded view of all the cell parts of modified AMPIX cell. (b)illustration of cross-sectional view of the cell when it is closed. (c) assembled in-house build modified AMPIX cell.

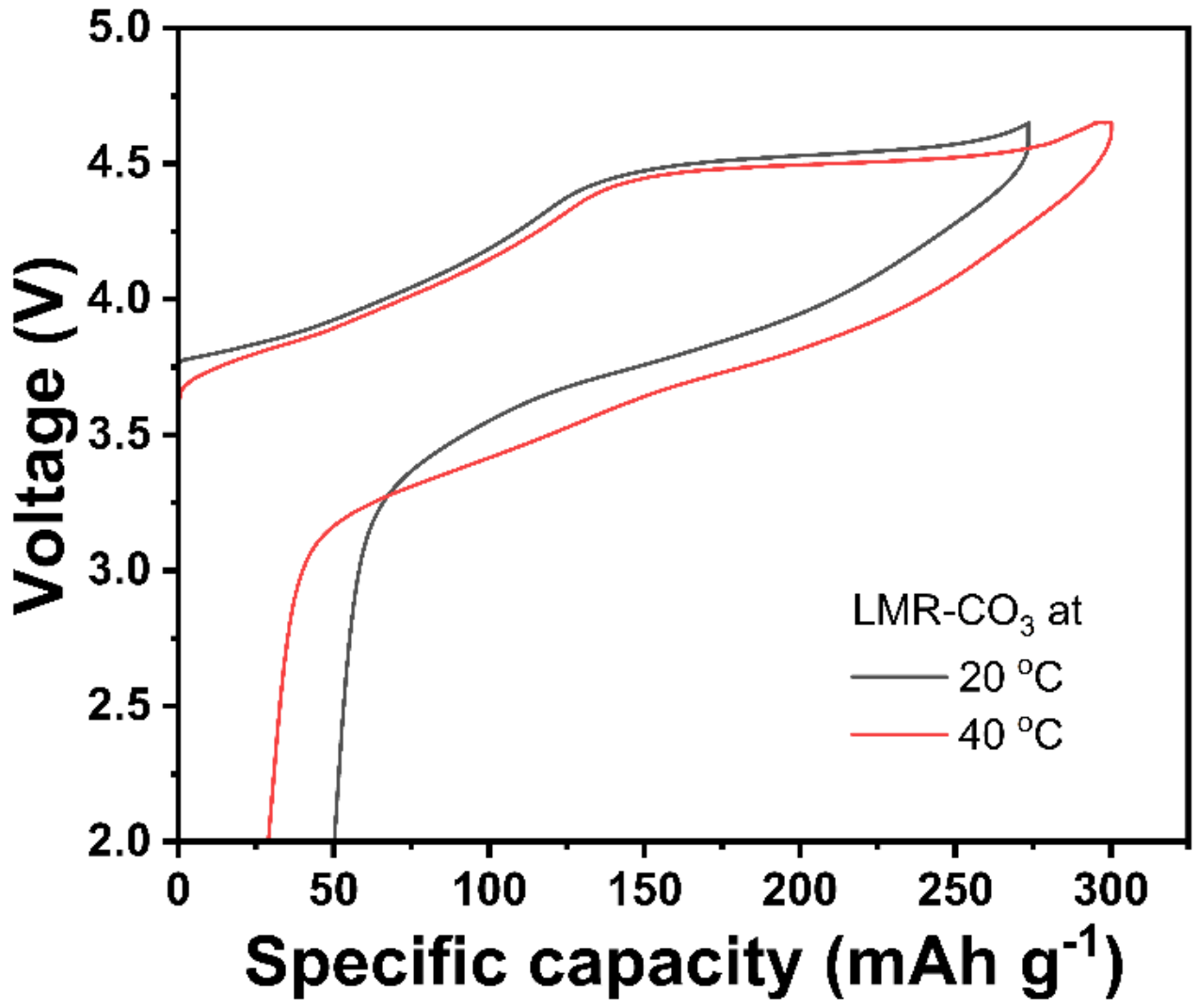

Figure S16: Voltage vs specific capacity profile of 1st cycle (activation cycle) done 20 °C and 40°C
